# UNified FramewOrk for reguLatory Dynamics (UNFOLD): Dissecting robustness, plasticity, evolvability and canalisation of biological function

**DOI:** 10.1371/journal.pcbi.1014289

**Published:** 2026-05-28

**Authors:** Debomita Chakraborty, Raghunathan Rengaswamy, Karthik Raman

**Affiliations:** 1 Bhupat and Jyoti Mehta School of Biosciences, Department of Biotechnology, Indian Institute of Technology (Madras), Chennai, India; 2 Centre for Integrative Biology and Systems medicinE (IBSE), IIT Madras, Chennai, India; 3 Department of Data Science and AI, Wadhwani School of Data Science and AI, IIT Madras,‌‌ Chennai, India; 4 Department of Chemical Engineering, IIT Madras,‌‌ Chennai, India; Indraprastha Institute of Information Technology Delhi, INDIA

## Abstract

A unique balance of seemingly contradictory properties like robustness and plasticity, or evolvability and functional canalisation, characterises biological systems. To understand the basis of these properties, we investigate gene regulation, which is at the core of biological function. We simulate dynamical models of over 190 million genetic circuits covering all possible three-gene circuit structures. Our computational pipeline classifies these circuits into functional clusters by matching their temporal response shapes. Thus, we generate a dataset linking circuit structure, parameters and a corresponding functional label. Our key finding is a finite list of 20 functions that three-node genetic circuits can perform under step input and within the explored parameter space. Moreover, the structure-parameter space for these circuits tends to be primed for responses that stabilise over time following a perturbation. Every structure exhibits potential for multifunctionality with a range of 2–17 functions contingent upon parameters. We quantify network degeneracy, showing that many structural changes can be made to circuits without altering function. We define three quantities: structural, parametric, and functional diversities. Using these diversities, we construct a UNified FramewOrk for reguLatory Dynamics (UNFOLD) to analyse four key biological properties—robustness, plasticity, evolvability, and functional canalisation. Using UNFOLD, and within the explored parameter space, we identify that only 6.5% of network structures are non-plastic, while parameter sets enabling parametric robustness exist for every three-node network. We identify functionally canalised circuits from structure pairs that can be interchanged for a large number of parameter sets without a change in function. Overall, our framework offers insights into the fundamental organisation of biological networks by thorough analysis of three-node networks.

## Author summary

Biological systems exhibit remarkable properties like robustness, plasticity, evolvability, and canalisation. This study presents a unified computational framework to understand these properties by exhaustively exploring the design space of three-node genetic circuits, identifying that only 20 functions are achievable, and revealing a bias toward stability. We uncover key principles of network degeneracy and multifunctionality, highlighting the versatility of genetic circuits. By analysing structural, parametric, and functional diversities, we characterise transitions between robustness, plasticity, canalisation, and evolvability. Our work advances theoretical insights into biological function and offers a structure-parameter-function map to identify alternate circuit designs and assess robustness, applicable when network structure and approximate parameter values are known from experiment or estimation.

## 1. Introduction

Biological systems demonstrate a fine balance of robustness, plasticity, evolvability and functional canalisation. Robustness is the ability of a system to maintain its function under changes in its structure and/or parameters, or under environmental changes. Over two decades ago, Wagner [1] suggested the possible bases of robustness in biological systems to be network structure and variability rather than redundancy introduced by duplicate genes. He elaborated on the coexistence of robustness and evolvability, noting that mutations can unleash evolutionary innovations by inching towards novel phenotypes, even when they do not affect a particular phenotype immediately, due to a neutral mutational space that confers mutational robustness to biological systems. The need for a unified framework with a strong mathematical basis to analyse biological robustness in all possible forms has long been recognised [2]. Evolvability may be defined as the potential to evolve or exhibit diverse phenotypes at the organism or population level. A comprehensive study of evolvability can be found elsewhere [3], along with methods to quantify this property from an evolutionary biology perspective. Plasticity is the property that allows organisms to tailor responses to internal or external cues dynamically. Waddington [4] coined the term canalisation to describe the low variance in phenotypes due to genetic and environmental variations in the wild-type, unlike strains of organisms manipulated in the laboratory.

In this work, we aim to construct a unified framework with a solid mathematical basis to analyse robustness, plasticity, evolvability, and canalisation at the level of gene regulation. For this, we consider a genetic circuit as a dynamical system whose response (“circuit function”) to a stimulus depends on the circuit structure and parameters [5–8]. We assume that mutations and epigenetic or environmental changes can affect gene regulation by changing the structure and/or parameters of genetic circuits, thereby influencing the circuit function, i.e., phenotype. In our unified framework, we do not directly quantify parametric robustness, plasticity, evolvability, and functional canalisation [9] but define conditions under which these properties are exhibited. These conditions are based on the changes in circuit structure and parameters. Our analysis does not refer to their underlying biological cause, which may be mutational, epigenetic, or environmental.

The space of all possible circuit structures and parameters constitutes the design space. We study the design space of all possible three-node genetic circuits based on the classic framework proposed by Ma *et al.* [10] and Shi *et al.* [11], who studied three-node networks to identify the structural conditions necessary for perfect adaptation. Our focus, however, expands to exploring the global design space of three-node genetic circuits to find all possible functions. Most existing works in the literature aim to design or fine-tune a genetic circuit to achieve only one functionality of interest. One notable work that delves into the design space is the Design Space Toolbox V3, which adopts a systems-theoretic approach [12,13]. While the Design Space Toolbox offers valuable insights, it has limitations that affect its applicability to our large-scale exploration. Specifically, it employs Generalised Mass Action (GMA) kinetics with power-law formulations. Although mathematically flexible, GMA kinetics presents challenges for systematic design space exploration at scale. First, GMA parameters—kinetic orders and rate constants—are phenomenological fitting parameters lacking direct biological interpretation, making it difficult to relate parameter values to underlying biochemical mechanisms. Second, GMA’s power-law formulation does not inherently enforce boundedness of gene expression levels, which can lead to numerical instabilities and biologically unrealistic unbounded growth during extensive parameter sampling across diverse network topologies. In contrast, we employ Hill kinetics, whose parameters–activation/inhibition thresholds, cooperativity, and degradation rates–correspond to measurable biochemical properties. Hill kinetics naturally incorporates saturation through its sigmoidal form, ensuring bounded responses that reflect biological reality where transcription factors cannot activate gene expression beyond maximal levels. This choice provides the right balance of biological realism, parameter interpretability, mathematical tractability, and numerical stability essential for reliably simulating and classifying 190 million circuits. [14]

We develop a computational approach to explore a broader scope than the previous works. Firstly, we map the circuit structures and parameters with the circuit functions. We simulate the mathematical models of the circuits over millions of parameter sets and pass the simulated data through our computational pipeline for clustering circuits with similar temporal responses. The output of this pipeline is a dataset with circuit structure and parameters, along with a functional label. Secondly, we use this labelled dataset to construct and use our unified framework for analysing the four biological properties– parametric robustness, plasticity, evolvability, and functional canalisation.

In Section 2, we describe our methodology for generating the simulated dataset and the computational pipeline we have used to process the dataset to get the labels. In Section 3, we discuss our key findings. Further details of our methodology and validation of our findings are provided in S1 Text.

## 2. Methodology

**Fig 1:**
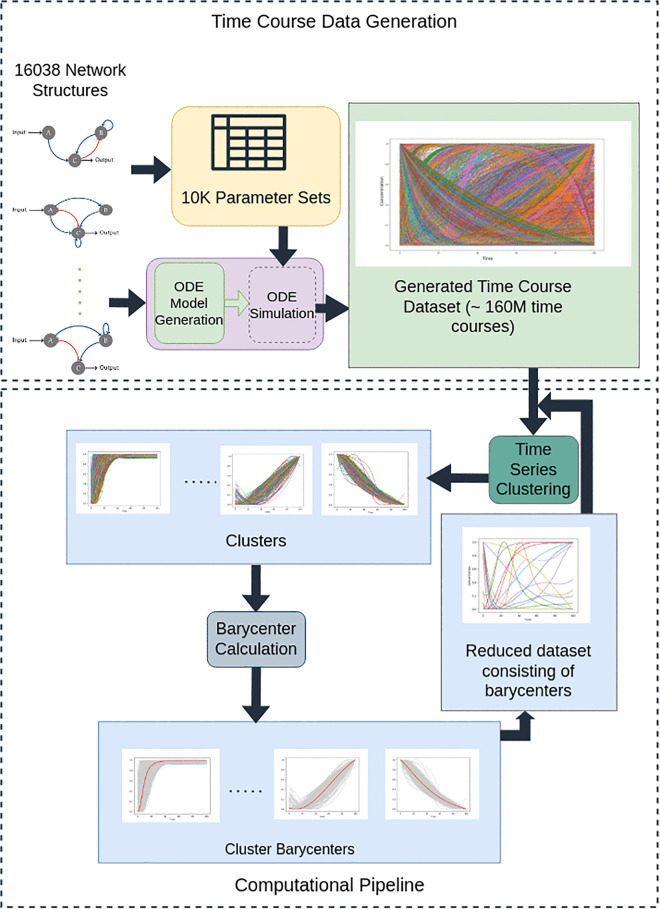
Workflow Diagram. We derive an ODE model for each of the 16,038 networks from their adjacency matrices and simulate these models over three sets of 10,000 parameter sets each, sampled using Latin Hypercube Sampling. We pass the resulting time-course dataset through a computational pipeline that performs time-series clustering, followed by barycenter calculation for each time-course cluster. This constitutes the first iteration of the pipeline. (The barycenter corresponding to a cluster is shown in red.) In every subsequent iteration,‌‌ the barycenters constitute a reduced dataset that serves as the input to the computational pipeline.

We develop a computational framework for studying the design space of genetic circuits. The main steps involved are generating time-course data by simulation and creating a computational pipeline for processing these data, as illustrated in Fig 1.

### 2.1. Simulation for time course data generation

**Fig 2:**
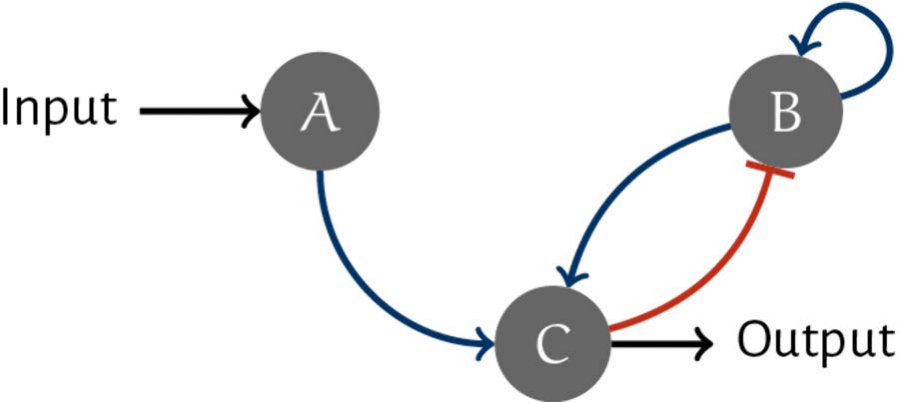
A representative three-node genetic circuit. *A*, *B*, *C* represent genes that interact by activation (shown as arrowheads) or inhibition (bar-heads). An activating input is applied to gene *A* while we study the expression of gene ***C*.**

We represent a genetic circuit as a network with genes as the nodes, as shown in Fig 2. Each gene in the network encodes a transcription factor (TF) that either activates or inhibits the expression of the other genes in the network. These two types of interactions are represented by two types of edges in the network, *viz.*, activation as arrowheads, and inhibition as bar heads. We label the three nodes as *A*, *B*, and *C*, where a step input is applied to node *A*; hence, it is designated the input node, while node *C* is the output node. The output gene expression over time for the given input, i.e., the expression of protein/TF from the output gene, *C*, is the focus of our analysis in this work. This approach is identical to that proposed by Ma [10].

#### 2.1.1. Network structure generation.

A three-node network is represented by a [3 × 3] adjacency matrix having three possible elements {−1,0,1}, representing inhibition, no interaction, and activation, respectively. Thus, the structure of all possible three-node networks can be obtained by generating all possible [3 × 3] adjacency matrices with {−1,0,1} as the only possible elements. There are 3^9^ = 19,683 such adjacency matrices/networks. Of the 19,683 networks possible, 3,645 networks have no direct or indirect connection going from the input to the output node. Our analysis is done on the remaining 16,038 networks [10,11].

#### 2.1.2. Sampling networks to obtain 10 partitions.

We systematically examine all possible three-node networks by partitioning the complete set of 16,038 networks into ten disjoint subsets through stratified random sampling without replacement. Each subset contains approximately 10% (1,604 networks) of the total, ensuring complete coverage where every network appears in exactly one partition. The stratification procedure ensures structural representativeness within each partition as follows: We first characterise the edge-type distribution across all 16,038 networks by calculating, for each of the nine positions in the 3×3 adjacency matrix, the frequencies of the three possible edge types: inhibition (-1), no interaction (0), and activation (+1) (Table B in S1 Text). We then employ stratified random sampling to divide the networks into ten subsets, ensuring that each partition maintains the same edge-type frequency distribution as the complete network collection. This guarantees that each partition captures the structural characteristics of the entire design space.

#### 2.1.3. ODE model generation.

We assume that Hill kinetics govern the interactions between the genes and the TFs, similar to the seminal study of Ma [10] and the subsequent work by Shi [11]. We generate the corresponding ODE models using the adjacency matrix for a network.


$$\begin{array}{c} \frac{dx}{dt}=v_{x}\left(\prod \frac{{\text{A}}_{i}^{n}}{{\text{A}}_{i}^{n}+K_{i}^{n}}\cdot \prod \frac{K_{j}^{n}}{{\text{I}}_{j}{\;}^{n}+K_{j}^{n}}\right)-\frac{x}{\tau_{x}} \end{array}$$ (1)

$$\begin{array}{c} \frac{dx}{dt}=\frac{1}{\tau_{x}}\left(\prod \frac{{\text{A}}_{i}^{n}}{{\text{A}}_{i}^{n}+K_{i}^{n}}\cdot \prod \frac{K_{j}^{n}}{{\text{I}}_{j}{\;}^{n}+K_{j}^{n}}\right)-\frac{x}{\tau_{x}} \end{array}$$ (2)

The general equation of a Hill kinetic model is shown in Eq. 1 where ${\text{A}}_{i}$ is the concentration of an activating gene product, ${\text{A}}_{i}\;\epsilon \;\{A,B,C,I\}$. ${\text{I}}_{j}$ is the concentration of an inhibiting gene product, ${\text{I}}_{j}\;\epsilon \;\{A,B,C\}$. The parameters involved in the equations are given below:

*v*_*x*_: maximal gene expression level for gene product *x*

*n*: cooperativity or Hill coefficient

$K_{i}/K_{j}$: activation/inhibition thresholds for the *i*-th activator/*j*-th inhibitor

$\tau_{x}$: half-life of gene product *x*

The activating and inhibiting interactions change the concentration of the gene product *x*, where xϵ{A,B,C}. As shown in Eq. 1, we assume an AND logic for the interactions, i.e., the gene *x* is activated only when all its activators are at high concentration and all its inhibitors are at low concentration [11].

#### 2.1.4. Parameter sampling.

Equation 1 has a maximum of 24 network parameters when a network has the maximum number of possible edges, i.e., nine edges. We transform Eq. 1 to Eq. 2 by non-dimensionalisation, and this reduces the maximum number of parameters for a network to 21 [11,15]. We use Latin Hypercube Sampling (LHS) to obtain 10,000 parameter sets from the 21-dimensional parameter space. We repeat this sampling three times to obtain three sets of 10,000 parameter sets each, which we name V0, V1, and V2. All the networks are simulated using the same super-set of 10,000 parameter sets. While simulating the ODE model of a network that does not have the maximum number of possible edges, the parameters corresponding to a missing edge are made zero. This is equivalent to sampling parameters separately for individual networks using LHS.

An activating step input (I) that changes from 0.06 to 0.6 at *t* = 0 is used for simulation. This input activates the input gene (*A*) with parameters $K_{I}=0.4$ and $n_{I}=1$ for all the networks simulated. Parameter ranges used for the simulation of ODE models are given below:

K:0.001−1 (sampled using a logarithmic scale)

n:1−4 (integral values sampled linearly)

τ:1−100 (sampled using a logarithmic scale)

#### 2.1.5. Initial conditions determination.

The initial conditions are determined by finding the steady states of a network for each of the 30,000 parameter sets with an input value of 0.06. A circuit is considered to have reached steady state only when all three gene products (corresponding to nodes A, B, and C) are stationary, not just the output node C. This stringent criterion is biologically appropriate because gene regulatory networks operate as coupled systems: a steady state in the output alone, while internal nodes continue to change, would not represent a true equilibrium of the circuit.

Two complementary approaches are employed to identify steady states for each parameter set. The primary method uses numerical solution for states where all derivatives equal zero, representing true mathematical equilibria. A candidate steady state is accepted if the derivative values for all three nodes are less than 10^−7^, ensuring that the system remains unchanged at that point. When numerical solution fails to converge to a valid steady state, either due to solver limitations or the absence of a mathematical equilibrium, a secondary empirical approach is implemented. This involves simulating the system dynamics over a sufficiently long time interval and examining changes in concentrations over the last six time points to determine whether the system has reached a practical steady state. The empirical criterion requires that the change in concentration for all three nodes across these final time points be less than 5%, indicating the system has stabilised sufficiently for practical purposes. This hybrid strategy ensures robust steady state identification by combining mathematical rigour with practical simulation-based verification, particularly valuable for complex or stiff systems where numerical solvers may struggle.

Of the potential 481,140,000 circuit simulations (16,038 networks × 30,000 parameter sets), only parameter sets for which a valid steady state exists and is greater than 0.001 for all three nodes are retained for further analysis. This filtering step removes circuits that do not converge to stable equilibria or exhibit unphysical steady states, resulting in approximately 190 million time courses that form the basis of our subsequent analysis.

### 2.2. Setting up a computational pipeline

We simulate the ODE models to obtain each network’s concentration dynamics for 100 time points over the parameter sets for which a steady-state greater than 0.001 could be determined. We construct a pipeline that recursively performs two operations: Time Series Clustering and Barycenter Calculation. We calculate the pairwise distances for time courses (min-max scaled) in our dataset using Dynamic Time Warping Distance (DTW-Distance) [16]. Subsequently, we perform K-Means clustering based on pairwise DTW distance. The number of clusters is determined by computationally locating the “elbow point” within a range of 2–100 clusters using the kneed library [17]. This automated approach is essential because our analysis requires determining the elbow point tens of thousands of times: once for each of the 16,038 networks in the first iteration of our computational pipeline, followed by clustering of pooled barycenter datasets in subsequent iterations, repeated across three versions of parameter sets (V0, V1, V2), totalling over 48,000 individual elbow point determinations. The kneed library provides a robust, reproducible, and objective mathematical method for elbow point detection by analysing the Within-Cluster Sum of Squares (WCSS) curve, eliminating the need for subjective visual inspection that would be impractical at this scale. For each cluster identified by K-Means, the barycenter is the time series representative of the cluster. We calculate the barycenters of each of the clusters using Soft-DTW [18].

In the first iteration, we cluster the time courses obtained for each network over 10,000 parameter sets. Suppose a cluster with fewer than 10 time courses is found; we drop these time courses from further iterations as these represent network functionalities that cannot be achieved by even 0.1% of the parameter sets [10]. For all the remaining clusters for each network, the barycenters are calculated. The set of barycenters across all the networks then forms a reduced dataset for the second iteration of time-series clustering. At the end of an iteration, we check if the shapes of all the barycenters are distinct, and if so, we terminate the execution at this iteration.

## 3. Results

**Fig 3:**
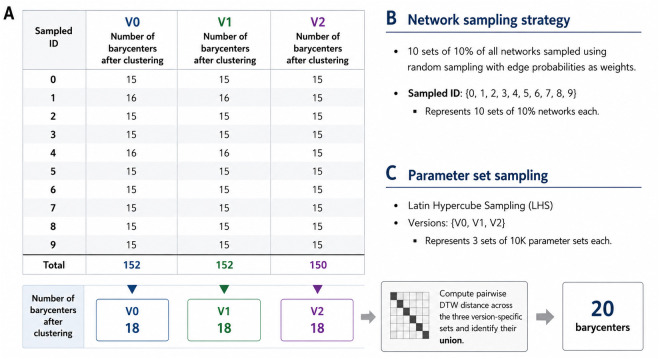
Summary of results. The 10 sampled sets of networks are labelled using the Sampled IDs from 0 to 9. The versions V0, V1, and V2 represent parameter sets. Each version contains 10,000 parameter sets.

We systematically examine all possible three-node networks by partitioning them into ten disjoint subsets as described in Section 2, with each partition comprising approximately 10% of the complete network set. This partitioning strategy serves two purposes: it makes the computational clustering tractable while enabling validation of our findings through independent analysis of structurally representative subsets. We run two iterations of our computational pipeline with the time courses for each partition of networks. In the second iteration, the reduced dataset consists of about 15–16 clusters. We observe this across all 10 partitions of networks as shown in Fig 3. To verify that the identified functions represent robust, reproducible dynamics rather than partition-specific artefacts, we pool the cluster barycenters from all 10 partitions and perform an additional clustering step to identify recurring function types across partitions. Furthermore, we repeat the complete application of our computational pipeline to three sets of 10,000 parameter sets (V0, V1, V2) each to check for consistency in the results obtained.

After all 10 partitions of networks are run through the computational pipeline, we collect the barycenters to form a combined dataset that we again run through our computational pipeline. After this step, we obtain 18 barycenters for each of the three versions of parameter sets. To determine if the 18 functions are similar or not, we calculate the pairwise DTW distances. If this distance is small, the corresponding pair of barycenters is very similar in shape and represents the same function. Based on this DTW distance, we then find the union of the 54 barycenters across the three versions. This results in 20 distinct barycenters, corresponding to the 20 circuit functions that three-node genetic circuits can perform.

### 3.1. Three-node networks perform 20 circuit functions

**Fig 4:**
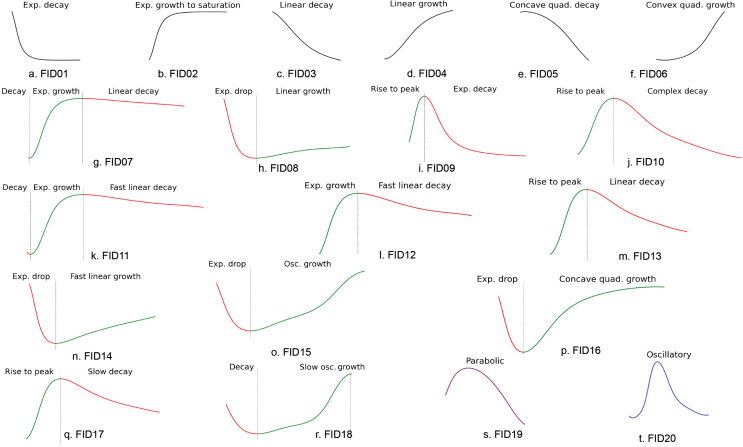
Three-node genetic circuit functions. The 20 distinct time courses characterise the functional space (in response to a step input) of all three-node genetic circuits.

**Fig 5:**
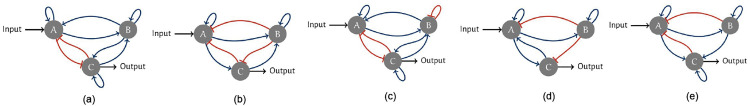
Network structures representing the most frequently observed functions. **(a)** FID01 - Exponential decay, **(b)** FID02 - Exponential growth to saturation, **(c)** FID03 - Linear decay, **(d)** FID04 - Linear growth, **(e)** FID05 - Quadratic decay - concave. These five functions collectively account for ~87% of all circuits. Each representative structure is determined by selecting the most frequent edge type (activation/inhibition/no interaction) at each position across all networks exhibiting that function.

Our analysis identifies a set of 20 functionalities achievable with three-node circuits over 30,000 parameter sets, detailed in Table 1. These represent distinct time courses, each embodying a unique shape that we interpret as a distinct circuit functionality, as illustrated in Fig 4. A noteworthy observation is that 17 out of 20 circuit functions consistently emerge across all three pipeline runs (Fig 3). Furthermore, we map each time course within a functional cluster to the specific network that generated it, thereby establishing a clear connection between the temporal dynamics and their underlying network structures. The descriptive names assigned to each function (Table 1) are qualitative labels based on visual inspection of barycenter curves, provided as convenient identifiers. We acknowledge that these names involve subjective interpretation, for example, “fast” vs. “slow” decay and “linear” vs. “sublinear” growth. We suggest referring directly to the time course plots in Fig 4 for interpretation. The key contribution is the computational identification of 20 distinct response patterns via unsupervised DTW-based clustering, not the specific nomenclature used to describe them. The representative network structures for the top five most observed functions (FID01–FID05) are shown in Fig 5. To identify a representative structure for a function, we aggregate all networks exhibiting that function and calculate the frequency of each edge type (activation/inhibition/no interaction) at each position in the [3 × 3] adjacency matrix. The representative structure selects the most frequent edge type at each position—effectively creating a consensus network that captures the dominant architectural features of that functional class. For instance, if the edge from A to B is an activation in 50% of networks showing FID01, an inhibition in 30%, and absent in 20%, the representative assigns an activation edge at that position.

**Table 1:** List of 20 functions that three-node genetic circuits exhibit. The distribution of circuits over the design space is not uniform, with most functions exhibited by <2% of the circuits while the most exhibited functions are observed in >25% of the circuits. Function names are qualitative descriptors; refer to Fig 4 for the actual time course dynamics.

Function ID	Function	% of circuits exhibiting the function
FID01	Exponential decay	28%
FID02	Exponential growth to saturation	27%
FID03	Linear decay	17%
FID04	Linear growth	11%
FID05	Quadratic decay - concave	3.2%
FID06	Quadratic growth - convex	2.4%
FID07	Decay followed by exponential growth followed by linear decay	2.3%
FID08	Exponential drop followed by linear growth	1.4%
FID09	Rise to peak followed by exponential decay	1%
FID10	Rise to peak followed by complex decay	1%
FID11	Rise to peak followed by linear decay	0.87%
FID12	Exponential growth followed by fast linear decay	0.82%
FID13	Decay followed by exponential growth followed by fast linear decay	0.75%
FID14	Exponential drop followed by fast linear growth	0.57%
FID15	Exponential drop followed by oscillating growth	0.45%
FID16	Exponential drop followed by concave quadratic growth	0.21%
FID17	Rise to peak followed by slow decay	0.19%
FID18	Decay followed by slow oscillating growth	0.05%
FID19	Parabolic	0.05%
FID20	Oscillatory	0.02%

### 3.2. The design space is primed to produce circuits that stabilise over time

Since circuit function depends on the network structure and the parameters governing the interactions within the network, we analyse the prevalence of the 20 functions exhibited by three-node networks in two ways: (a) in terms of network structure alone (network distribution) and (b) in terms of network structure with parameters defined (circuit distribution). In Table 1, we show the high occurrence of circuits showing exponential decay (28%) and the circuits that saturate after exponential growth (27%) compared to the other circuit functions. This disparity suggests that the design space favours some functions over others and aligns intuitively with the observed tendency of biological systems to navigate toward stabilisation. The least frequent functions are characterised by oscillations (FID20), a parabolic trajectory in time (FID19), and decay followed by oscillating growth (FID18). We note that the percentage of oscillatory networks represents only networks that are initially at a steady state and only respond to a step input with oscillations. Since we have eliminated any circuit that inherently shows instability (including oscillations) when we determine the initial conditions for our models, this may explain the small percentage of oscillatory networks. Moreover, we have eliminated any behaviour exhibited by a network for less than 10 out of 10,000 parameter sets in the first iteration of our computational pipeline. These eliminated time courses may include oscillatory as well as adaptive behaviours. We separately analysed the simulated time courses to look for adaptive networks using criteria from existing works [10,11]. The structural analysis of the adaptive networks reveals that using Hill kinetics, it is possible to achieve adaptation in three-node networks even without negative feedback loops or incoherent feedforward loops, unlike previously reported results by Shi [11]. The detailed findings on adaptive networks are given in Section 1.2 in S1 Text.

### 3.3. Every network is multifunctional

A notable observation in our analysis of three-node genetic circuits is that none of the networks displayed mono-functional behaviour. As we change the parameters of a given circuit, showing a particular function, it produces at least one new function at a different point in the parameter space. This finding highlights the intrinsic versatility of gene regulatory networks, showcasing their ability to exhibit diverse functionalities under varying parametric conditions. The number of distinct functions a three-node network performs spans an impressive range, from at least two to as many as 17 functions.

We further categorise the 20 circuit functions into five categories, *viz.*, (i) monophasic, (ii) biphasic, (iii) triphasic, (iv) oscillatory, and (v) complex based on the number of phases a time response can be divided into.

Fig A in S1 Text shows detailed distributions of the networks over the function categories and allows a nuanced analysis of the design space. The percentage of circuits that exhibit the functions corresponding to each function category is much smaller than that of networks that fall in the corresponding category. This underscores the pivotal role of parameters in biological regulation, as they determine a network’s function and complement its structural characteristics. For instance, while 5% of networks are capable of oscillatory behaviour, merely 0.02% of circuits exhibit purely oscillatory dynamics (FID20)—sustained oscillations in response to the step input starting from a stable steady state. This excludes circuits with oscillatory features superimposed on other trends, which we classify as “complex” dynamics. All 16,038 networks are capable of exhibiting monophasic responses. However, networks that exclusively exhibit monophasic dynamics constitute only 21.8% of all networks. Interestingly, 53% of the networks can exhibit all the categories of functions under different parametric conditions except for oscillations, suggesting that an oscillatory response to a step input is a rare function. Other combinations of function categories are exhibited by <5% of the networks, with the rarest combinations being monophasic (I) and oscillatory (IV), or monophasic (I), biphasic (II) and oscillatory (IV). The rarest category of circuit function is oscillatory, while more than 90% of the circuits exhibit monophasic responses.

### 3.4. Network degeneracy allows a large number of structural changes with no change in function

We define network degeneracy as a measure of structural changes (edge addition/removal/change in the sign of an edge) that preserve function when parameters remain constant. Specifically, we quantify: for a given parameter set and function, how many different network structures can produce that same function with that exact parameter set. This directly measures the ability to make structural modifications without functional consequences when parameters are held fixed.

Our methodology leverages the 21-dimensional parameter space used for all 16,038 networks (Section 2). Each network is simulated using parameter sets from this 21D space, with parameters corresponding to absent edges set to zero. When comparing networks that differ by edge addition or removal, we use the same underlying 21D parameter set; the only difference is which parameters are active (non-zero) versus inactive (zero). For edge sign changes (activation ↔ inhibition), we maintain the same threshold (K) and cooperativity (n) parameter values but reverse the regulatory effect in the ODE model. This approach allows us to directly test how many structurally distinct networks produce the same function given a fixed parameter set, thereby quantifying network degeneracy: the number of structural variants that are functionally equivalent under identical parametric conditions.

**Fig 6:**
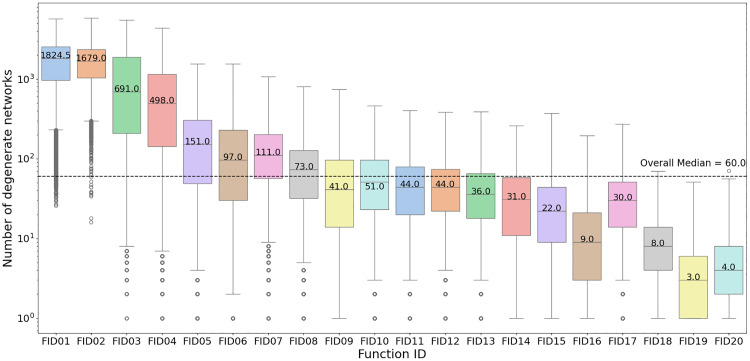
Network degeneracy across different functions. The median number of degenerate networks across all functions is 60, indicating that there can be 60 different structures with at least one addition/removal of an edge, with/without a change in the sign of edges (and parametric changes corresponding to only the added/removed edge, with other parameters remaining unchanged) without a change in function. We find that the median network degeneracy varies widely for different functions.

Fig 6 shows the median number of structurally distinct networks over all parameter sets for each of the 20 functional clusters. While network degeneracy depends on the function under consideration, we find a median of 60 structural variants across all functions. This means that, on average, one can make substantial structural modifications to a circuit without losing its function, provided the parameters for unchanged edges remain constant, and parameters for new/removed edges follow our sampling scheme.

### 3.5. A UNified FramewOrk for reguLatory Dynamics (UNFOLD)

**Fig 7:**
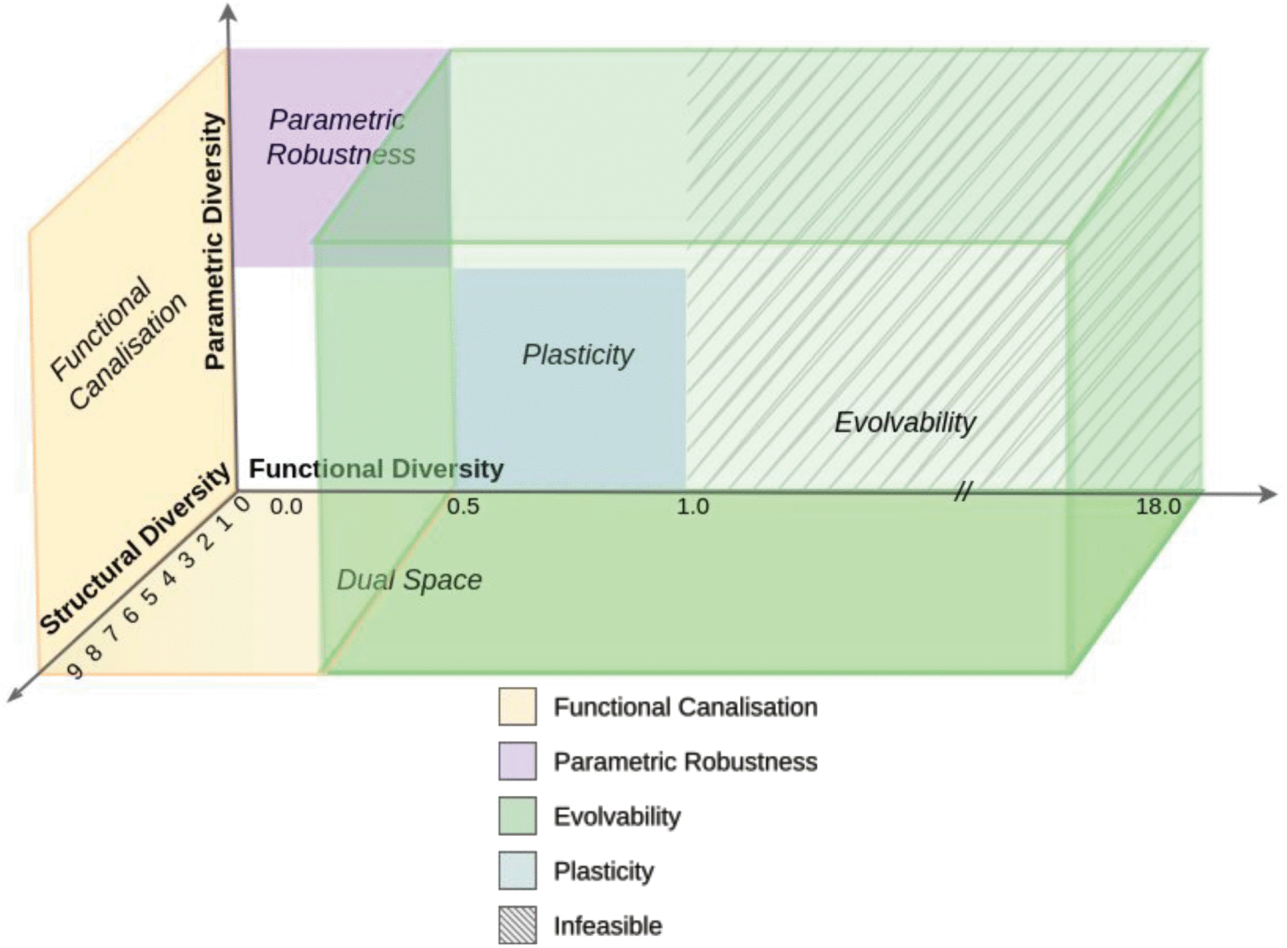
A UNified FramewOrk for reguLatory Dynamics (UNFOLD). A conceptual framework for quantifying conditions for parametric robustness, plasticity, functional canalisation, and evolvability in terms of structural, parametric and functional diversities.

We now describe a conceptual framework, UNFOLD, that unifies the analysis of the four properties of biological systems—parametric robustness, plasticity, evolvability and functional canalisation—using the results of our computational pipeline, as shown in Fig 7. Considering a pair of circuits *C*_*i*_ and *C*_*j*_ with structures, parameter sets and functions given by (*G*_*i*_, *P*_*i*_, *f*_*i*_) and (*G*_*j*_, *P*_*j*_, *f*_*j*_), respectively, we define three pairwise distance measures that quantify dissimilarity in structure, parameters, and function: structural diversity (SD), parametric diversity (PD), and functional diversity (FD). We use “diversity” to emphasise the degree of variation in each dimension, consistent with terminology in evolutionary and systems biology.

These diversity measures were designed to enable systematic analysis of biologically meaningful properties across our 190-million-circuit dataset while being: (1) computationally tractable for large-scale pairwise comparisons, (2) biologically interpretable, and (3) aligned with our goal of identifying discrete regions in the conceptual space corresponding to specific biological properties.

**Structural diversity** (*SD*_*ij*_) between *C*_*i*_ and *C*_*j*_ is quantified by the Hamming distance between the adjacency matrices *G*_*i*_ and *G*_*j*_ and assumes 10 discrete levels [0−9].

**Parametric diversity** (*PD*_*ij*_) is quantified by the Euclidean distance between parameter sets *P*_*i*_ and *P*_*j*_ in the 21-dimensional parametric space. While alternative distance metrics could be considered, Euclidean distance effectively captures the magnitude of parametric changes while maintaining computational tractability and interpretability. For circuits sharing the same network structure (*SD* = 0), we verified that the coefficient of variation for pairwise parametric distances exceeds 0.466 for all 16,038 networks, indicating sufficient diversity in our dataset to avoid dimensionality-related artefacts.

**Functional diversity** (*FD*_*ij*_) quantifies the evolutionary/mutational distance between circuit functions, unlike the DTW distance, which measures time-series similarity in our clustering pipeline. We employ a discrete formulation for FD because it: (1) enables clear boundaries in the conceptual space (Fig 7) that precisely define biological properties (e.g., FD = 0 demarcates functional canalisation), (2) aligns with biological conceptualisations of functional similarity, and (3) enables efficient computational querying of biologically relevant regions. FD is calculated using equation 3 and comprises two components:


$$\begin{array}{c} FD_{ij}=HD_{k-hot}+w_{ij}HD_{1-hot} \end{array}$$ (3)

The structural component ($HD_{k-hot}$) measures differences in the functional repertoires of network structures. We encode each network function as a *k*-hot binary vector of length 20 corresponding to the list of 20 possible functions with *k* indicating the number of functions exhibited by the network and ranging between 2−17. The Hamming distance between the *k*-hot function vectors for *G*_*i*_ and *G*_*j*_ gives $HD_{k-hot}$ which captures how structural differences influence multifunctionality.

The parametric component ($w_{ij}HD_{1-hot}$) measures categorical distance between specific circuit functions. We encode each circuit function as a 20-bit one-hot binary vector. $HD_{1-hot}$ is the Hamming distance between these function codes (0 if *f*_*i*_ and *f*_*j*_ are the same; else, 1). The weight *w*_*ij*_ quantifies the biological significance of functional transitions based on dynamical complexity differences between function categories of *f*_*i*_ and *f*_*j*_ (Table D in S1 Text), where $w_{ij}\in \{0,0.5,1.0\}$ reflects increasing differences in dynamical complexity. This scheme reflects the principle that greater differences in dynamical complexity correspond to larger functional distances, although qualitative patterns remain consistent across reasonable variations in weight. FD is a discrete variable assuming 37 unique values ranging between 0−18. For circuit pairs that share the same network structure ($SD_{ij}=0$ since $G_{i}=G_{j}$), $HD_{k-hot}=0$, so FD can assume only 3 values {0.0, 0.5, 1.0}. This shows that constraining the structural diversity also constrains the functional diversity.

Using our computational pipeline, we have functionally labelled over 190 million three-node genetic circuits. Each data point in the conceptual space in Fig 7 involves the calculation of structural, parametric and functional diversities for pairwise circuits. The number of computations required to analyse all labelled circuit pairs is highly time-consuming and resource-intensive. We identify four properties of biological systems that can be analysed in terms of the three diversities: functional canalisation, parametric robustness, plasticity, and evolvability. These can be mapped to specific regions of the conceptual space in Fig 7. Hence, we confine our analysis to these biologically relevant regions of this space and derive insights that will help us advance our understanding of the design space. The representation in Fig 7 allows us to abstract the effect of mutations and/or epigenetic changes as changes in genetic circuit structure and/or parameters and enables us to dissect the role of structure and parameters in producing functional canalisation, parametric robustness, plasticity, and evolvability, in a unified framework.

Our analysis operates at circuit-level resolution, where every data point represents a specific circuit pair: (*G*_*i*_, *P*_*i*_, *f*_*i*_) and (*G*_*j*_, *P*_*j*_, *f*_*j*_). We compute structural, parametric, and functional diversities for these pairs and summarise the findings with ensemble statistics for interpretability. While our discrete sampling approach (30,000 parameter sets per structure) approximates but does not fully capture continuous parameter space structure, this approach is biologically appropriate for modelling mutational and epigenetic changes, which cause discrete jumps in parameter space rather than continuous drift.

#### 3.5.1. Conditions for parametric robustness and plasticity.

Suppose mutations or epigenetic changes lead to changes in parameters but not the circuit structure; the circuits before and after the change constitute a pair with $SD_{ij}=0,PD_{ij}\neq 0$. For all such pairs of circuits, we calculate the parametric and functional diversity and find the overall mean parametric diversity $PD_{overall\_mean}$.

**Parametric Robustness:** We identify circuit pairs demonstrating parametric robustness, defined as maintenance of function despite parametric changes exceeding the ensemble mean. Specifically, for all circuit pairs where $SD_{ij}=0$ (same structure) and $PD_{ij}\neq 0$ (different parameters), we consider pairs with $FD_{ij}=0$ and $PD_{ij}>PD_{overall\_mean}$ to exhibit this property. A circuit (*G*_*i*_, *P*_*i*_, *f*_*i*_) is parametrically robust if there exist other parameter sets *P*_*j*_, *P*_*k*_, ... such that (*G*_*i*_, *P*_*j*_, *f*_*i*_), (*G*_*i*_, *P*_*k*_, *f*_*i*_), ... all maintain function *f*_*i*_ despite *PD*_*ij*_, $PD_{ik}>PD_{overall\_mean}$.

**Plasticity:** Circuit pairs that have $FD_{ij}\neq 0$ and $PD_{ij}<PD_{overall\_mean}$ are considered plastic, as they exhibit diverse functions with relatively small changes in parameters. This indicates sensitivity to parametric perturbations, where below-average parameter changes can lead to functional transitions.

**Fig 8:**
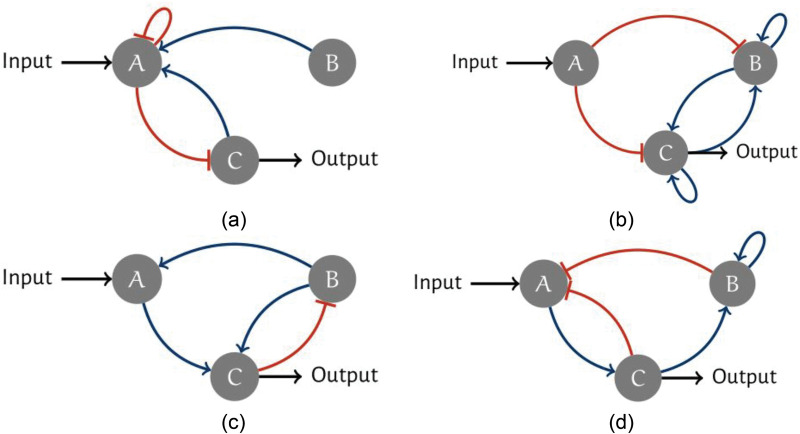
Structures of most robust and plastic circuits. **(a)** The most common structure among all robust circuit pairs. **(b)** The structure that produces the highest percentage (52%) of robust circuit pairs out of all the circuit pairs that share this structure. **(c)** The most common structure among all plastic circuit pairs. **(d)** The structure that produces the highest percentage (40%) of plastic circuit pairs out of all the circuit pairs that share this structure is shown.

We analysed the relationship between circuit structure and parametric robustness/plasticity in two ways for data points with zero structural diversity (SD). First, we identified the most frequently occurring network structures among robust and plastic circuit pairs. These predominant structures are illustrated in Figs 8a and 8c for robust and plastic circuits, respectively. Second, we quantified the propensity of each network structure to generate robust or plastic behaviour, Fig 8b. The most striking example, shown in Fig 8d, exhibits robustness in 52% of all circuit pairs sharing this structure. We found 209 distinct network structures maintaining robust behaviour across more than half of their parameter sets. In contrast, even the most plastic-prone structure exhibits parameter-dependent behaviour in only 40% of cases. Notably, our analysis revealed a fundamental asymmetry: while every three-node network structure can achieve robust behaviour with appropriate parameter selection, 1,043 structures (6.5% of the total) never display plastic behaviour, regardless of their parameters.

#### 3.5.2. Conditions for functional canalisation and evolvability.

Suppose a circuit undergoes structural and parametric changes ($SD_{ij}\neq 0,PD_{ij}\neq 0$) due to mutations or epigenetic change but retains its function; then the circuit pair before and after the change represent a data point on the *FD* = 0 plane. To ensure $PD_{ij}\neq 0$, we consider structural changes with at least one edge being added or removed (more details in Section 1.3 in S1 Text). A nuance to consider in this context is that when a mutation leads to a change in the structure of a circuit from *G*_*i*_ to *G*_*j*_, it inherently leads to changes in parameters corresponding to the edge changes, while the parameters corresponding to the unaltered edges may or may not be affected. In our analysis, we do not distinguish between the two scenarios. Instead, we track only whether the circuit pair has zero functional diversity ($FD_{ij}=0$).

**Functional Canalisation:** We interpret every point on the *FD* = 0 plane to be functionally canalised since they represent circuits that can maintain functionality even after changes in structure and parameters (Fig 7). When a circuit pair having structures that share the same function vector ($HD_{k-hot}=0$) exhibits the same function ($HD_{1-hot}=0$), or it shows two distinct functions, but of the same category ($w_{ij}=0$), the functional diversity is zero (Eq. 3). We find that canalised circuit pairs tend to have a peak SD between 4 and 6, indicating that a medium level of structural diversity is conducive to canalisation, as shown in Fig B in S1 Text.

**Fig 9:**
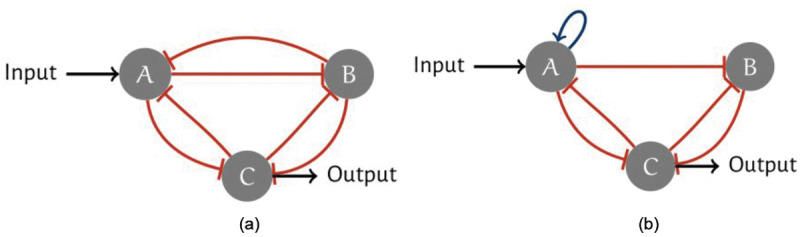
Functional canalisation illustrated through the toggle triad. **(a)** The toggle triad topology studied by [19] exhibits 14 distinct functions across our explored parameter space. **(b)** A structurally different network (Hamming Distance = 2) produces the identical 14-function repertoire across parameter space, demonstrating functional canalisation where both structural and parametric changes can occur while preserving the functional repertoire. This example illustrates how well-characterised circuits, such as the toggle triad, are not unique but represent points within a broader landscape of functionally canalised architectures.

To illustrate functional canalisation with a concrete example, we examined the well-studied toggle triad topology [19]. Our analysis reveals that the toggle triad exhibits 14 distinct functions across the explored parameter space, demonstrating substantial multifunctionality. Remarkably, we identified another network structure differing from the toggle triad by only two edges (Hamming Distance = 2) that produces the identical 14-function repertoire across parameter space (Fig 9). This represents functional canalisation because both structural changes (the two-edge difference) and parametric changes (different parameter sets for each structure) can occur while the functional repertoire remains preserved. Table 2 shows the distribution of parameter sets over the 14 functions produced by the toggle triad and its equivalent network. This example demonstrates that well-characterised circuits, such as the toggle triad, are not unique in their functional capabilities but rather represent specific implementations within a broader landscape of functionally canalised architectures. Such canalised circuit pairs provide reliable alternate design options for achieving the same functional repertoire in synthetic biology applications, where robustness to both structural perturbations (mutations) and parametric variations (expression level changes) is desirable.

**Table 2:** Percentage distribution of parameters over functions. The 14 functions produced by the toggle triad network and its equivalent network are given along with the percentage of the parameter sets (total 8813 for the toggle triad and 6951 for the toggle triad equivalent circuit) that produce each of these functions.

Function ID	Percentage of Parameters (Toggle Triad)	Percentage of Parameters (Toggle Triad Equivalent)
FID01	36.32	38.79
FID02	8.74	9.73
FID03	22.6	23.49
FID04	7.99	9.61
FID06	9.95	7.41
FID08	2.38	1.19
FID09	2.04	1.02
FID10	2.3	2.1
FID11	1.97	1.78
FID12	1.51	1.55
FID13	1.32	2.42
FID14	1.08	0.35
FID15	0.87	0.33
FID18	0.92	0.23

**Evolvability:** Circuit pairs for which $FD_{ij}\neq 0$ and $SD_{ij}\neq 0$ are defined as evolvable since they represent circuit pairs with both structural and parametric changes leading to new functions. The space representing evolvability with FD≠0 is shown in Fig 7.

The dual space encompasses the special case where $PD_{ij}=0$. We discuss this space in detail in Section 1.3 in S1 Text.

## 4. Discussion

Our study presents a comprehensive exploration of the three-node genetic circuit design space, analysing over 190 million circuits to reveal fundamental principles of biological regulation. This unprecedented scale of analysis has led to discoveries that reshape our understanding of biological circuit design and function. While computational models have long enabled synthetic biological circuit design [20,21], previous approaches like Tang and co-workers’ study of perfect adaptation [10] or Chiang’s KMFA pipeline focused on optimising circuit parameters for single functionalities [22]. Our work transcends the scope of these previous works by providing a complete map of the achievable functional space and establishing a unified framework for understanding circuit behaviour.

The discovery that only 20 distinct functions are achievable by three-node genetic circuits in response to step input perturbations represents a fundamental insight into the constraints of biological regulation. This finding, which emerged from analysing 30,000 parameter sets across all possible three-node network structures, suggests an inherent limit to the complexity achievable with three-node circuits under these conditions. Remarkably, the distribution of these functions is highly non-uniform, with over 55% of circuits exhibiting either exponential decay or exponential growth to saturation. This strong bias toward stabilising responses to perturbation suggests a natural tendency for regulatory systems to return to equilibrium over time. The implications of this finding extend beyond theoretical interest; it provides crucial guidance for synthetic biology efforts by defining the complete space of achievable functions with three-node circuits.

Our analysis revealed two fundamental properties of genetic circuits with profound implications for both natural and synthetic systems. First, every network structure examined exhibits multifunctionality, capable of producing between 2 and 17 distinct functions depending on parameter configurations. This universal multifunctionality is a constraint imposed by the physics of transcriptional regulation under Hill kinetics, defining the feasibility space for three-node genetic circuits. While we cannot conclude that natural circuits evolved to exploit this versatility without direct evidence from comparative genomic data, our framework establishes the boundaries of what is achievable and provides testable predictions about evolutionary selection pressures (discussed below). Our finding of approximately 60 median degenerate structures per function means that mutations causing structural changes have a high probability of being neutral (maintaining function), creating the possibility for neutral evolution within functional classes that can be tested through phylogenetic analysis of actual circuits. This significant network degeneracy, where multiple structural changes often preserve function without parameter adjustments, has immediate practical applications in synthetic biology, offering multiple design options for achieving desired functions and suggesting strategies for engineering robust circuits.

The unified framework we developed, based on structural, parametric, and functional diversity metrics, provides a computational approach to analysing these biological properties systematically. Previous studies by von Dassow [23] quantified robustness of the segment polarity gene function through the count of random parameter samples that produce a phenotype. Wagner [24] suggested an antagonistic relationship between genetic robustness and evolvability but a positive correlation between phenotypic robustness and evolvability due to large neutral spaces, indicating a many-to-one map between genotype and phenotype. However, Mayer showed using Boolean maps between genotype and phenotype that Wagner’s suggestion was valid only in special cases, while in general, phenotypes are more likely to have a trade-off between robustness and evolvability [25]. UNFOLD moves away from the genotype-phenotype paradigm to the circuit level to simultaneously consider structural, parametric, and functional aspects. This approach revealed that while every three-node network can achieve robust behaviour with appropriate parameters, 6.5% of structures are fundamentally non-plastic: a finding that is significant for synthetic biological applications where plasticity is not a desired property.

While well-characterised examples like the *lac* operon [26] and *kai* circadian genes [27] provide important functions arising from the interaction of three genes, our work reveals a much broader landscape of possible circuit behaviours. The practical relevance of our findings extends to critical areas like cancer research, as exemplified by the RAS gene family (*KRAS*, *HRAS*, and *NRAS*) [28,29]. Our framework provides new ways to characterise the functional landscape of such three-gene systems within the complete feasibility space. We note, however, that applying the UNFOLD diversity measures to a specific biological circuit requires knowledge of the underlying network structure and parameter values, information that must be obtained from experimental data or parameter estimation. Subject to this prerequisite, our framework can inform the prioritisation of structural or parametric perturbations as candidate therapeutic interventions, by identifying which changes are likely to shift circuit function versus which are buffered by robustness or canalisation. Our approach complements experimental methods such as scRNA-seq and ATAC-seq by providing a forward-design perspective rather than inferring networks from data. Our data repository serves as a valuable resource for synthetic biology, offering a comprehensive map of structure-parameter-function relationships and enabling rational design of circuits with desired properties. This is particularly powerful when combined with our understanding of network degeneracy and alternative implementations, as it allows designers to choose optimal implementations based on practical constraints.

Several exciting avenues exist for expanding this work. While we focused on step input responses, future investigations could explore other input types to determine whether additional functions emerge with different perturbation patterns, and incorporate noise, which has been shown to produce richer dynamics [22]. Extending the parameter space exploration could also reveal additional functional categories or alter the distribution of observed functions. Our DTW-based clustering approach proved effective for identifying the 20 distinct functions observed in three-node circuits, successfully distinguishing responses across diverse complexity levels: simple monotonic dynamics (exponential decay, growth to saturation), biphasic responses (growth-then-saturation, decay-then-growth), triphasic responses (FID07, FID13), and complex behaviours (FID10, FID15, FID18). The consistency of identifying 17 of 20 functions across three independent parameter set runs (Fig 3), including subtle multiphasic distinctions, validates the method’s robustness for three-node circuit complexity. Since all time courses were normalised to [0,1] prior to DTW clustering, circuits with identical temporal shape but differing response amplitudes receive the same functional label. While this normalisation was appropriate for our goal of classifying qualitative response shapes, future work could apply DTW to unnormalised trajectories, or use multivariate DTW incorporating amplitude as an explicit dimension, to resolve finer amplitude-based distinctions within functional categories where signal magnitude is biologically relevant. However, larger networks may generate increasingly complex responses with subtle distinctions that challenge trajectory-based similarity metrics, where functionally important differences could be obscured by overall shape similarity. Empirical validation with larger network simulations would determine where complementary approaches become necessary, such as feature-augmented clustering combining DTW with frequency-domain and dynamical features, multi-scale DTW capturing patterns at different temporal resolutions, or hierarchical clustering using DTW for broad categorisation followed by feature-based refinement for subtle within-category distinctions. We also note that while we worked with Hill kinetics, other kinds of regulatory mechanisms, such as post-translational modifications and RNA-level regulation, would require different modelling frameworks, although our computational pipeline can be used as long as the processing involves time-course data.

While our work provides complete functional enumeration and structure-function mapping, detailed mechanistic analysis of how specific regulatory paths and motifs produce each function remains important for future investigation. Such analysis would require systematic decomposition of input-to-output paths, classification of coherent versus incoherent motifs, and examination of parameter-dependent path dominance—a substantial undertaking given that each function is produced by hundreds to thousands of network structures with parameter-dependent dynamics.

The framework could be extended to larger networks. While exhaustive exploration becomes computationally intractable for larger networks due to combinatorial explosion (the number of possible structures grows as approximately $3^{n^{2}}$ for n-node networks with three edge types), our study establishes methodological approaches that can be scaled to larger systems. A key insight is that our weighted sampling strategy successfully captures functional diversity, as partitioning the 16,038 three-node networks into ten independent subsets (each 10%) consistently identifies 15–16 functional clusters across all partitions (Fig 3). This consistency demonstrates that strategic sampling of larger network spaces can effectively capture the predominant functional repertoire, enabling machine learning approaches for design space characterisation.

Specifically, functionally labelled datasets from sampled network subsets can train graph neural networks (GNNs) for semi-supervised learning of functional labels. GNNs naturally encode network topology through message-passing architectures, can learn meaningful representations of regulatory motifs and structural patterns, and enable the prediction of functional labels for unsampled network structures based on topological similarity. Our discovery that every network exhibits multifunctionality (Section 3.3) directly informs the formulation of such problems: functional label prediction should be cast as a multi-label graph classification problem (predicting the repertoire of functions a network structure can exhibit across parameter space) rather than a single-label problem. This semi-supervised strategy would involve: (a) applying our computational pipeline to a strategically sampled, tractable subset of larger networks, (b) training GNN models on the resulting structure-parameter-function dataset, and (c) predicting functional labels for the vast majority of unsampled networks, thereby characterising the broader design space without exhaustive simulation.

Our discrete sampling approach provides dense coverage of parameter space but does not map continuous robustness boundaries around individual circuits. While our analysis characterises the feasibility space for three-node circuits under Hill kinetics, it does not directly address evolutionary selection pressures or historical trajectories. However, as discussed above, the framework enables testing whether evolution has preferentially selected for robust and/or evolvable designs by comparing natural circuits against our mapped feasible space. Future work combining our framework with comparative genomic analyses of natural three-node circuits could test whether observed circuits occupy regions predicted to be rare (suggesting positive selection) or common (suggesting drift), and whether they cluster in robust versus plastic regions of the design space. Such analyses would transform our feasibility mapping into direct insights about evolutionary processes. Our findings also suggest specific experiments to validate the predicted rare functional categories and test the practical implementation of canalised circuits.

The key contribution of this study goes beyond providing a conceptual framework; it reveals universal properties such as multifunctionality and network degeneracy and offers practical guidelines for circuit design. By unifying the analysis of robustness, plasticity, evolvability, and canalisation, we bridge the gap between theoretical understanding and practical application, opening new avenues for both basic research and synthetic biology ‌‌applications.

### Code Sharing

All the code used for the current work is publicly available at https://doi.org/10.5281/zenodo.15387089.

## Supporting information

S1 Text Table A: Adaptive networks having different structures across the three sets of parameters given by V0, V1, and V2. Table B: Edge probabilities table. Probabilities of different categories of edges occurring in the complete set of 16,038 networks. Table C: Function Categories. The 20 functions that three-node networks perform can be categorised based on the number of phases into which a given time response can be divided. Table D: Weights table for calculation of functional diversity. The rows and columns represent the categories as given in Table C. We further sub-categorise the monophasic category (I) into I.A and I.B representing decay and growth, respectively. Sub-category I.A includes functions FID01, FID03, and FID05 while I.B includes FID02, FID04, and FID06. Fig A: Upset plots for network and circuit distributions over function categories. Fig B: Distribution of canalised circuit pairs over SD.
(TEX)
