# Supplementary material for: UNified FramewOrk for reguLatory Dynamics (UNFOLD): Dissecting robustness, plasticity, evolvability and canalisation of biological function
Source: PLoS Comput Biol. 2026 May 28;22(5):e1014289. doi: 10.1371/journal.pcbi.1014289 (PMC13252850; doi:10.1371/journal.pcbi.1014289)
Supplement: Attachment — Submitted filename: Consolidated Rebuttal Letter.pdf [file pcbi.1014289.s003.pdf]

Dear Prof. Ahuja,

We would like to thank you and the reviewers for your thoughtful and constructive feedback on our manuscript titled 'UNified FramewOrk for reguLatory Dynamics (UNFOLD): Dissecting Robustness, Plasticity, Evolvability and Canalisation of Biological Function'. We appreciate the opportunity to revise and resubmit the work.

We have carefully considered all the comments raised by the reviewers and have made substantial revisions to the manuscript in response. Below, we provide a point-by-point response to each comment. Reviewer comments are reproduced in red, followed by our responses in regular text. Changes made in the manuscript are indicated with sections (as applicable).

We believe that the manuscript has been significantly improved as a result of the reviewers' constructive input. We hope that the revised version is now suitable for publication in *PLOS Computational Biology*.

We sincerely thank you for your time and consideration.

Sincerely,
Debomita Chakraborty, Raghunathan Rengaswamy and Karthik Raman

## Reviewer 1

The manuscript introduces a computational framework titled "UNified FramewOrk for reguLatory Dynamics (UNFOLD)", which seeks to systematically classify and elucidate the dynamic behaviors of gene regulatory networks (GRNs) under diverse conditions and structural properties. This work addresses an important and timely challenge in systems biology—understanding how different network topologies and parameter configurations give rise to distinct dynamic regimes. The approach adopted by the authors is both novel and conceptually significant, as it provides a unified methodology for analyzing and categorizing GRNs based on their dynamical characteristics rather than solely on their structural features. By integrating these aspects, UNFOLD offers a valuable tool for researchers aiming to interpret the functional implications of GRN dynamics. It is commendable that the authors have done an extensive job in analysing such a large number of networks. However, the biological insights from this exercise are largely lacking, and I request the authors to address these points:

Major comments --

1) Can the authors comment on key biological insights from their analysis? Which set of networks give rise to which properties? Please present at least some examples in the high-dimensional network topology and parameter space where a particular behavior can be mapped to a set of networks, irrespective of the parameters? Similarly, it is not clear how pairwise comparisons between circuits would help to define a global conceptual space for these properties. For instance, the authors should comment on whether the properties seen by a toggle triad (Duddu et al. J R Soc Interface 2020) are seen in any other 3-node network, and if yes, how do those topologies compare to toggle triad?

We thank the reviewer for this insightful question about the biological insights and specific examples of network-property relationships. We address each point below:

Our analysis reveals several fundamental biological insights about three-node genetic circuits:

1. Universal multifunctionality as a physical constraint: Every network structure we examined exhibits multifunctionality, capable of producing between 2 and 17 distinct functions depending on parameter configurations. This universal multifunctionality is a constraint imposed by the physics of transcriptional regulation under Hill kinetics, defining the feasibility space for three-node genetic circuits. This has profound implications: natural circuits occupying rare regions of our mapped space would suggest positive selection for specific dynamics, while clustering in robust versus plastic regions would reveal whether evolution has favoured robustness or evolvability.
2. Network degeneracy enables neutral evolution: Our finding of approximately 60 median degenerate structures per function means that mutations causing structural changes have a high probability of being neutral (maintaining function), creating the possibility for neutral evolution within functional classes. This can be tested through phylogenetic analysis of actual circuits.
3. Asymmetry between robustness and plasticity: While every three-node network can achieve robust behaviour with appropriate parameters, 6.5% of structures are fundamentally non-plastic—a finding that is significant for synthetic biological applications where plasticity is not a desired property.
4. Bias toward stability: The design space exhibits a strong bias toward stabilising responses to perturbation, with over 55% of circuits exhibiting either exponential decay or exponential growth to saturation. This suggests a natural tendency for regulatory systems to return to equilibrium over time.

To address the reviewer's concern about presenting specific examples, we have made significant additions to the main text:

- We now present the structural representatives (Fig. 5) for the top 5 most frequently observed functions (FID01-FID05), which collectively account for 87.87% of all circuits. Each function is exhibited by multiple network structures. To identify a representative structure for each function, we aggregate all networks exhibiting that function and calculate the frequency of each edge type (activation/inhibition/no interaction) at each position in the 3×3 adjacency matrix. The representative structure selects the most frequent edge type at each position—effectively creating a consensus network that captures the dominant architectural features of that functional class.
- The adjacency matrices for structural representatives of all 20 functions are provided in the Supplementary Information, enabling readers to examine the full spectrum of structure-function relationships.

The reviewer asks how pairwise comparisons define a global conceptual space. This reflects a critical aspect of our methodology that we clarify here:

Our analysis operates at circuit-level resolution, where every data point represents a specific circuit pair: ($G_i$, $P_i$, $f_i$) and ($G_j$, $P_j$, $f_j$). We compute structural, parametric, and functional diversities for these pairs and summarise the findings with ensemble statistics for interpretability. This approach provides several advantages:

1. While individual pairwise comparisons provide fine-grained information about specific circuits, the ensemble statistics reveal global patterns. For example, in Section 3.5.1, we analyse all circuit pairs where $G_i = G_j$ (same structure, $SD_{ij} = 0$) but $P_i \neq P_j$ (different parameters, $PD_{ij} \neq 0$). For each such pair, we determine whether $f_i = f_j$ (function preserved) or $f_i \neq f_j$ (function changed). The ensemble view reveals that every three-node network can achieve robust behaviour with appropriate parameters, while 6.5% of structures never display plastic behaviour.
2. Consider a specific network structure (e.g., mutual inhibition topology). In our dataset, we have ~30,000 different parameter sets producing ~30,000 circuits with this structure. We can identify, for instance, that circuit $C_1 = (G_1, P_1, f_3)$ and circuit $C_2 = (G_1, P_2, f_3)$ maintain the same function despite parametric distance $PD_{ij} = 5.2$. It is possible to trace all circuits within any defined PD radius around $C_1$ and determine which maintain $f_3$ (constituting a "robust neighbourhood") and which switch functions (defining the boundary of robustness).
3. While our discrete sampling approach (30,000 parameter sets per structure) approximates but does not fully capture continuous parameter space structure, this approach is biologically appropriate for modelling mutational and epigenetic changes, which cause discrete jumps in parameter space rather than continuous drift.

The reviewer raises an excellent question about the toggle triad studied by Duddu et al. (J. R. Soc. Interface, 2020). We note an important methodological distinction: Duddu et al. analysed

the stable states of the toggle triad network at steady state, whereas our work applies a step input perturbation to networks and analyses the temporal dynamics (time courses) in response to that input. These are complementary but distinct analytical frameworks.

Despite this methodological difference, we have identified important connections:

1. In our analysis, the toggle triad produces 14 of the 20 functions identified in our study across the parameter space we explored. This demonstrates the exceptional multifunctionality of this well-studied circuit topology.
2. We identified another network structure that produces the same 14-function repertoire as the toggle triad. Remarkably, this network differs from the toggle triad by only two edges (Hamming Distance = 2), providing a concrete example of network degeneracy—substantial structural changes that preserve the functional repertoire.
3. We have included a new figure (Figure 9) that shows both the toggle triad and its degenerate structural variant side by side, explicitly illustrating how two-edge modifications preserve the functional capabilities. This serves as a compelling example of the structure-property relationships the reviewer requested.

This analysis demonstrates that the properties observed in well-characterised circuits, such as the toggle triad, are not unique but rather represent points within a broader landscape of functionally equivalent or similar network architectures. Our framework enables systematic identification of such relationships across the entire three-node design space.

We have substantially enhanced the presentation of network-property relationships by:
(1) adding structural representatives for the top 5 functions to the main text,
(2) providing adjacency matrices for all 20 functions in Supplementary Information,
(3) including a specific comparison with the toggle triad and a structural variant with equivalent functions, and
(4) clarifying how pairwise circuit-level analyses aggregate to reveal global ensemble properties.
These additions directly address the reviewer's request for concrete examples mapping network topologies to specific behaviours and properties.

2) It can get computationally intractable to extend this study to larger networks, but how would insights from this be helpful in characterizing the properties of larger networks?

We thank the reviewer for this important question about the scalability and broader applicability of our framework to larger networks.

We acknowledge that exhaustive exploration of all possible network structures becomes computationally intractable for larger networks due to the combinatorial explosion of possible

topologies. For n-node networks with three possible edge types (activation, inhibition, or no edge), the number of possible structures grows as $3^{n^2}$, making brute-force enumeration infeasible beyond three nodes. However, our study provides several insights and methodological approaches that can be leveraged for characterising larger networks:

A key finding from our work is that our weighted sampling strategy successfully captures the functional diversity of the complete network space. We partitioned the 16,038 three-node networks into ten independent subsets (each 10% of the total), using edge-probability-weighted sampling, and demonstrated that each subset consistently identified 15-16 functional clusters (Figure 3). This consistency across structurally representative partitions suggests that strategic sampling of larger network spaces, rather than exhaustive enumeration, can effectively capture the predominant functional repertoire. The functionally labelled dataset generated by our computational pipeline for a sampled subset of larger networks can serve as training data for machine learning approaches. Specifically, we propose using graph neural networks (GNNs) for semi-supervised learning of functional labels. GNNs are particularly well-suited for this task because they:

i. Naturally encode network topology through message-passing architectures

ii. Can learn meaningful representations of regulatory motifs and structural patterns

iii. Enable prediction of functional labels for unsampled network structures based on topological similarity

Importantly, our discovery that every network exhibits multifunctionality (Section 3.3) directly informs the formulation of such machine learning problems, specifically, by casting functional label prediction as a multi-label graph classification problem (predicting the repertoire of functions a network structure can exhibit) rather than a single-label problem, when not considering specific parameter values. This semi-supervised approach would allow us to: (a) apply our computational pipeline to a strategically sampled, computationally tractable subset of larger networks, (b) train GNN models on the resulting structure-parameter-function dataset, and (c) predict functional labels for the vast number of unsampled networks, thereby characterising the broader design space without exhaustive simulation.

Our comprehensive three-node network analysis identifies fundamental regulatory motifs and their associated functions. These motifs serve as building blocks in larger networks. Understanding how three-node subcircuits contribute to function provides a foundation for decomposing and analysing larger networks as compositions of well-characterised smaller modules.

The UNFOLD framework, based on structural, parametric, and functional diversity metrics, provides conceptual tools applicable to networks of any size. While we cannot exhaustively

explore all larger networks, we can apply these metrics to compare specific larger networks of biological interest, analyse how larger networks exhibit robustness, plasticity, evolvability, and canalisation, and identify functionally canalised alternatives for specific biological circuits.

We have added a discussion of these scalability considerations and future directions to the revised manuscript (in the Discussion section).

3) The main text and figures do not adequately present what kind of network designs give the particular properties mentioned. The authors have mainly referred to these in supplementary figures, while the network properties are the primary focus of this study. Thus, they should consider better visualization of their key observations as main figures.

We have added a comprehensive figure (Figure 5) showing representative network structures for the top 5 most frequently observed functions (FID01-FID05), which collectively account for 87.87% of all circuits, directly illustrating how different network topologies produce specific functional behaviours.

Each function is exhibited by multiple network structures. To identify a representative structure for each function, we aggregate all networks exhibiting that function and calculate the frequency of each edge type (activation/inhibition/no interaction) at each position in the 3×3 adjacency matrix. The representative structure selects the most frequent edge type at each position—effectively creating a consensus network that captures the dominant architectural features of that functional class. For instance, if the edge from A to B is an activation in 50% of networks showing FID01, an inhibition in 30%, and absent in 20%, the representative assigns an activation edge at that position.

We have also moved the illustration for structures that are most robust and plastic from the Supplementary Figures section to the main text. These changes ensure that network architecture-property relationships are prominently featured in the main manuscript, addressing the reviewer's concern about adequate presentation of our key structural findings.

4) The authors have used LHS sampling in 21-dimensional space and reduced the dimension when not all parameters are required. However, since LHS is not independent across dimensions, can the authors justify that reducing the dimensions would maintain the necessary properties?

We thank the reviewer for this important methodological question. We address this concern through our implementation approach, mathematical justification, and rigorous validation.

Our methodology uses a unified sampling strategy that inherently preserves LHS properties:

Single 21D LHS for all networks: We generate parameter samples in the full 21-dimensional space (3 degradation rates, 9 activation/inhibition thresholds, and 9 cooperativities for all possible edges in a three-node network) using Latin Hypercube Sampling. Critically, all 16,038 network structures use the same 21D LHS samples—for any given parameter set, every network receives the same underlying 21-dimensional vector.

Network-specific parameter zeroing: For each network, parameters corresponding to absent edges are set to zero, while parameters for present edges retain their sampled values. This is implemented in the '*make_unnecessary_params_zero*' function in our code.

*Example*: Network A with edges A→B, B→C uses parameters {$K_{AB}$, $n_{AB}$, $K_{BC}$, $n_{BC}$} from the 21D vector (others set to zero), while Network B with edges A→C, C→B uses {$K_{AC}$, $n_{AC}$, $K_{CB}$, $n_{CB}$} from the same 21D vector (others set to zero).

Our approach sets parameters to zero, which is mathematically equivalent to marginalising those dimensions. When LHS is performed in *d* dimensions and *k* dimensions are subsequently set to zero, the remaining (*d-k*) dimensions maintain their LHS stratification properties. This is because LHS ensures that each dimension is independently stratified into n equal-probability intervals, with one sample per interval. Setting some dimensions to a constant value does not affect the stratification of the remaining dimensions, which preserve the space-filling and coverage properties of LHS.

To rigorously validate this approach, we performed Kolmogorov-Smirnov goodness-of-fit tests (detailed in Supplementary Section S1.1, which includes the validation code and complete results).

Our test procedure is given below:

1. Generated 10,000 samples in 3D using LHS, then set z=0 to obtain 2D samples (mimicking our parameter zeroing)
2. Independently generated 10,000 samples directly in 2D using LHS
3. Compared marginal distributions using two-sample K-S tests
4. Repeated for 1D (setting y and z to zero)

K-S test p-values = 1.0 for all coordinates at both dimensionalities.

The K-S test compares empirical cumulative distribution functions of two samples. A p-value of 1.0 indicates the maximum distance between CDFs is minimal—the distributions are essentially identical. This result is expected and reassuring: LHS, by design, creates uniform marginal distributions, so proper marginal projection should yield distributions indistinguishable from native-dimension LHS. Our p-value = 1.0 confirms this theoretical expectation.

Beyond statistical tests, our results demonstrate adequate parameter space coverage:

- Consistency across runs: Three independent runs with 10,000 parameter sets each yielded consistent results (17 of 20 functions emerged across all runs; Supplementary Section S1.6)
- Consistency across network partitions: Analysis of 10 structurally diverse network partitions showed consistent functional clusters (15-16 clusters across all partitions)

This consistency would not occur if our sampling approach had systematic coverage gaps. Our unified 21D LHS approach with network-specific parameter zeroing is mathematically sound (equivalent to marginal projection), statistically validated (K-S tests confirm preserved distributions), and empirically robust (consistent results across runs and partitions). We appreciate the reviewer's attention to this technical detail, which has allowed us to clarify the rigour of our methodology.

5) Page 8, Line 146: When it comes to 20 distinct behaviours observed in the 3-node networks, how easy or difficult is it to identify such behaviors in larger systems where the complexity of the response can increase? Is the method scalable to more complicated types of responses too?

We thank the reviewer for this important question about whether DTW-based clustering can effectively distinguish increasingly complex response types in larger networks. Our analysis demonstrates DTW's effectiveness across diverse complexity levels in three-node circuits:

- Simple monotonic responses (exponential decay, growth to saturation)
- Biphasic responses (growth-then-saturation, decay-then-growth)
- Triphasic responses (such as FID07, FID13)
- Complex dynamics (FID10, FID15, FID18)

The successful identification of 20 distinct functions, including subtle differences between multiphasic responses, and consistency across three independent runs (17 out of 20 functions, Fig. 3), indicates that DTW effectively handles considerable complexity for three-node circuits. However, we acknowledge that larger networks may produce responses with subtle distinctions requiring complementary methods. Empirical validation with simulated data from larger networks would be necessary to confirm effectiveness or identify where hybrid approaches are needed, for instance, if complex responses become increasingly similar in overall shape. DTW may assign similar distances to qualitatively different responses; subtle but functionally important differences may be obscured by overall trajectory similarity. If DTW proves insufficient for more complex responses, we propose complementary approaches: (1) feature-augmented clustering combining DTW with frequency-domain characteristics and dynamical features, (2) multi-scale DTW capturing features at different time resolutions, (3) hierarchical clustering using DTW for broad categorisation, then feature-based refinement for subtle distinctions.

We have added a discussion of these considerations and validation analysis to the revised manuscript (Section 4).

6) Page 12, Line 224: When the authors mention structural diversity, as the Hamming distance between two circuits, how would the Hamming distance be calculated? Are the circuits aligned such that they give the lowest or highest hamming distance between the adjacency matrices? Would it not be better to use some kind of isomorphism measure between the circuits, as it would be more robust than just the hamming distance between two circuits?

We thank the reviewer for this important question about our structural diversity metric. We clarify our approach and explain why graph isomorphism measures are not applicable to our study. Structural diversity (SD) between two circuits is calculated as the Hamming distance between their adjacency matrices. Each 3×3 adjacency matrix is flattened into a 9-element vector, and the Hamming distance is computed as the number of positions where the two vectors differ. All networks in our study have three nodes with fixed functional roles: node A is the input node (receiving the step stimulus), node C is the output node (whose expression we monitor), and node B is an intermediate node. This fixed labelling establishes a canonical representation: position [i,j] in any adjacency matrix always represents the directed edge from node i to node j. Therefore, there is no ambiguity requiring alignment. Graph isomorphism measures are designed for unlabeled graphs where node identities can be permuted. However, our networks have labelled nodes with distinct functional roles that cannot be interchanged. Additionally, our adjacency matrices encode both edge presence and regulation type (activation/inhibition), making each position biologically meaningful and non-permutable.

For our labelled, directed networks, Hamming distance is the appropriate metric because:

1. Biological interpretability: Each unit of Hamming distance represents one edge addition, removal, or sign change, which are directly interpretable as a mutational change
2. No information loss: Preserves all biologically relevant distinctions between circuits
3. Computational efficiency: Enables analysis of millions of circuit pairs

Minor comments --
1) Is n assumed to be the same for all regulations in equation (1)?

No, the Hill coefficient (n) is not assumed to be the same for all regulatory interactions in Equation (1). In our model, each of the nine possible edges in a three-node network has its own independently sampled Hill coefficient.

Specifically, for a three-node network with nodes A, B, and C, there are nine possible regulatory edges (A→A, A→B, A→C, B→A, B→B, B→C, C→A, C→B, C→C). Each edge in Equation (1) has its own

Hill coefficient which is sampled independently from the range [1-4] using Latin Hypercube Sampling.

2) While the authors have checked for connectedness from input to output, can they also check if the connectedness is satisfied between all edges to eliminate cases like A->B and A->C, where node B does not affect the output?

In this work, we aimed to examine all networks that have a direct or indirect edge connecting the input node to the output node. Thus, any cases where A->B and A->C edges exist, but B does not affect the output, were also a part of the analysis. We considered this a valid case since the criterion that there should be a direct/indirect edge from the input (A) to the output (C) is satisfied, even though B does not take part in the regulation of C directly; its presence diverts some of the regulatory effect of A from reaching C. We essentially consider such networks to be valid three-node networks.

3) Could the core functions also be explained in terms of the effective edge from input to output, separating the case with coherent/incoherent edges as well?

We thank the reviewer for this insightful question about analysing core functions in terms of effective input-to-output paths and distinguishing coherent versus incoherent feed-forward motifs. This represents a future direction for a deeper mechanistic understanding.

Our work establishes the necessary foundation by: (1) identifying all 20 achievable functions, (2) mapping which structures produce which functions, (3) providing consensus architectures for each function (main text for top 5; Supplementary Information for all 20), and (4) developing quantitative metrics (SD, PD, FD) for systematic comparison. Future work can leverage this foundation to systematically address coherent/incoherent path analysis for functions of specific biological or synthetic interest.

Our manuscript presents an exhaustive enumeration of 16,038 structures, ~190 million circuit simulations, characterisation of 20 functions, the UNFOLD framework, and analysis of robustness, plasticity, evolvability, and canalisation. Adding a comprehensive structural decomposition of all 20 functional clusters would exceed the scope of a single publication.

Each of the 20 functions is exhibited by hundreds to thousands of distinct network structures. For example, the most common functions (FID01: exponential decay and FID02: exponential growth to saturation) are each produced by over 98% and 90% of the 16,038 networks, respectively. A thorough structural analysis would require:

1. Systematic path decomposition: Identifying all direct and indirect paths from input to output, classifying them as coherent or incoherent, and determining which path combinations are necessary for observed dynamics.
2. Parameter-dependent path dominance: The same network structure produces multiple functions depending on parameters (Section 3.3). Effective regulatory paths depend critically on kinetic parameters (thresholds K, cooperativities n, time constants τ), not topology alone. A complete analysis would require mapping how parameter variations shift dominance between competing regulatory paths.
3. Emergent network-level effects: Three-node networks exhibit complex dynamics that cannot be reduced to simple input-output paths. Our analysis revealed that adaptation can be achieved without canonical negative feedback loops or incoherent feedforward loops (Supplementary Information Section S1.2), indicating that emergent dynamics resulting from network-level interactions play a crucial role in this process.

This analysis constitutes a distinct research problem requiring:

- Motif enumeration and classification algorithms
- Dynamical systems and control theory approaches
- Comparative structural analysis frameworks to identify essential versus dispensable architectural elements

We have added a note in the Discussion identifying this as valuable future work.

4) This statement is not clear: "The percentage of circuits that exhibit the functions corresponding to each function category is much smaller than that of networks that fall in the corresponding category."

While many network structures have the potential to produce functions in a given category (depending on parameters), far fewer individual circuits (specific structure-parameter combinations) actually exhibit those functions. For example, although 5% of network structures can produce oscillatory behaviour, only 0.02% of circuits (structure-parameter pairs) actually exhibit oscillations.

5) Page 8, Line 152: Can the parametrised Jacobian of these networks be used as a precursor for deciding which networks are stable and which would not be stable? As the time after which a network will reach the steady state is highly dependent on the parameters, having an independent method of cross-checking would make the classification more accurate. If that is something which would not be computationally scalable, can the simulations not be run for a longer time to determine if the system indeed shows oscillations?

We thank the reviewer for this insightful suggestion regarding the use of Jacobian analysis. We agree that Jacobian analysis at steady states is a valuable complementary approach. However, for our classification purposes, there are several considerations:

1. The Jacobian eigenvalues are indeed parameter-dependent, which means stability classifications would vary across parameter space. Our goal is to classify network topologies based on their *capacity* for different behaviours across biologically relevant parameter ranges, rather than stability at specific parameter values.
2. For 16038 network topologies, each sampled at 30,000 parameter sets, computing and analysing Jacobians at all steady states would require additional computational cost. However, we acknowledge this could provide valuable validation for a subset of networks.
3. Linear stability analysis (Jacobian) may miss complex nonlinear phenomena such as limit cycles that are stable but don't appear as stable fixed points, or basin-dependent dynamics where multiple attractors coexist.

Regarding the parameter dependence of the steady state of a network, we agree with the reviewer and describe below how we have already applied the method proposed by the reviewer.

Two complementary approaches were employed to identify steady states for each parameter set. The primary method used is ‘fsolve’ to numerically solve for states where all derivatives equal zero, representing true mathematical equilibria. A candidate steady state was accepted if the derivative values for all three output nodes were less than $10^{-7}$, ensuring the system would remain unchanged at that point. When ‘fsolve’ failed to converge to a valid steady state, either due to solver limitations or the absence of a mathematical equilibrium, a secondary empirical approach was implemented. This involved simulating the system dynamics over the specified time interval (with extension of this time by up to 10 times) and examining the change in concentrations over the last six time points to determine if the system had reached a practical steady state. The empirical criterion required that the change in concentration for all three output nodes across these final time points be less than 5%, indicating the system had stabilised sufficiently for practical purposes. This hybrid strategy ensured robust steady state identification by combining mathematical rigour with practical simulation-based verification, particularly valuable for complex or stiff systems where numerical solvers may struggle.

6) Page 11, Line 205: “We define network degeneracy as a measure of structural changes (edge addition/removal/ change in the sign of an edge) that can be made, with no subsequent parameter adjustment, without causing loss of function.” Wouldn’t the removal or the change in the sign of the edge lead to a completely different ODE structure? And instead of the parameter becoming zero, the ODE itself will not have that parameter? Could the authors please clarify this section of the results a bit better?

We clarify both the methodology and address the reviewer's correct observation about ODE structure changes.
Edge removal, addition, or sign change does lead to a different ODE structure. We do not simply set parameters to zero within a single fixed ODE; rather, we derive and simulate the specific ODE corresponding to each network structure. Each of the 16,038 network structures has its own ODE model derived from its adjacency matrix (Section 2). A network with edge B→C has a different ODE than a network without this edge—the regulatory term involving B's effect on C is present in one ODE and absent in the other. When comparing networks with different structures, we are comparing results from different ODEs, not the same ODE with different parameter values. Despite having different ODEs, our methodology enables a systematic comparison across network structures. All 16,038 networks are evaluated using the same 30,000 parameter sets, sampled in a 21-dimensional space (corresponding to all possible parameters for fully connected three-node networks). For each of the 30,000 parameter sets, we:

1. Simulate each network structure with its specific ODE, using the relevant parameters from the 21D set
2. Determine which networks reach steady state and what function each exhibits
3. Count how many structurally different networks (with their corresponding different ODEs) produce the same function when using the same 21D parameter set (with each ODE using only its relevant parameters)

This represents a biologically meaningful scenario: a structural mutation occurs (changing which genes interact or how they interact), and the biochemical properties (binding affinities, cooperativities, degradation rates) encoded in the parameters remain unchanged. The question is whether this structural change, with fixed biochemical properties, alters the circuit's function. When we change an edge sign, we simulate the modified ODE with the same K and n parameter values (from the 21D set) but in the appropriate regulatory context (activation vs. inhibition term).

7) Why would the clustering give some non-overlapping set of barycenters across parameter versions? This implies that the clustering is not robust across parameter replicates and opens up the possibility that there might be more than 20 circuit functions given enough sampling. Another possibility is that this could be due to the <10 time series filtering criteria.

The reviewer correctly identifies that the three version-specific functions suggest incomplete parameter space sampling. However, these rare functions (FID18, FID19, FID20) collectively account for only 0.12% of all circuits, while the 17 consistently appearing functions represent 99.88% of circuits (Figure 3, Table 1).

The high consistency (17 out of 20 functions across all versions) demonstrates that our sampling captures the predominant functional landscape. The three rare functions likely represent:

1) Functions requiring specific parameter fine-tuning (<0.1% of parameter space)
2) Edge cases near dynamical transitions
3) Behaviours filtered by our <10 time series criterion (intentionally excluding functions in <0.1% of parameter sets)

We acknowledge the reviewer's valid point that additional sampling could reveal more functions. However, the diminishing returns (only 3 additional rare functions across 30,000 parameter sets, representing 0.12% of circuits) suggest we have approached saturation of the commonly accessible functional space. Any additional functions would likely be increasingly rare and potentially biologically inaccessible due to parameter noise.

8) It is not clear why sampling of networks is performed (although it seems more like partitioning). Is it so that the clustering algorithm can process the data in chunks?

The reviewer is correct that this is more accurately described as "partitioning" rather than random sampling. We employed this partitioning strategy rather than analysing all networks together for two reasons:

1. Computational efficiency in clustering: Our computational pipeline performs iterative time-series clustering using DTW distance calculations. Processing all ~480 million time courses (16,038 networks × ~30,000 parameter sets) simultaneously would be computationally prohibitive. Partitioning reduces the clustering problem into manageable chunks while preserving the ability to identify all function types through the subsequent meta-analysis.
2. Validation of function discovery: By analysing ten independent, structurally representative subsets, we can verify that the functions we identify are not artefacts of a particular network sample. If the same functions emerge consistently across multiple independent partitions, this provides confidence that we have captured the true functional landscape.

9) Page 5, Line 64: "Of the 19683 networks possible, 3645 networks have no direct or indirect connection going from the input to the output node. Our analysis is done on the remaining 16038 networks."
Would the analysis have been easier to do if only the non-isomorphic networks from these networks had been chosen? That would also ensure that in the meta-analysis, the reported number of behaviour

s would not have been biased by the number of isomorphic networks present in the networks considered.

Graph isomorphism is not applicable to our network set because our three-node networks have fixed, functionally distinct labels: specifically, node A is the input node (receiving perturbation), node C is the output node (monitored expression), and node B is the intermediate node. These functional roles are fundamental to our analysis and cannot be permuted.

Because nodes have fixed, non-interchangeable roles, networks that would be isomorphic in an unlabeled graph context represent biologically distinct circuits in our framework. Therefore, all 16,038 networks in our dataset are functionally distinct, with no redundancy from isomorphic equivalents. Our comprehensive analysis of all connected networks accurately captures the complete design space of three-node genetic circuits with a defined input-output architecture. Furthermore, our meta-analysis is unbiased, as it does not suffer from the over-representation of structurally equivalent networks.

10) Page 12, Line 226: Euclidean distance has been shown to capture very little for larger dimensions. Could the authors comment on whether this behavior is avoided in their implementation or is inconsequential for the analysis? If not, could the authors please address how the methodology can be scaled to larger networks?

We thank the reviewer for bringing this point to our attention. Euclidean distance in the 21-dimensional parameter space provides a straightforward measure of parametric changes. While other distance metrics (such as Manhattan and Mahalanobis) could be considered, Euclidean distance effectively captures the magnitude of parametric changes in the parameter space. We chose simplicity and interpretability over more complex metrics. Furthermore, we have checked a subset of our dataset specifically containing the parametric diversity among circuits that share the same structure, i.e., all circuit pairs that fall on the SD = 0 plane. For a given network, we considered the pairwise distance between all circuits sharing this network structure and calculated the coefficient of variation. We found that for each of the 16038 networks, the coefficient of variation is at least 0.466. This indicates that our dataset for analysing robustness (SD = 0) is not affected by the curse of dimensionality. We acknowledge that to avoid the curse of dimensionality, alternative distance metrics can be explored in future work to quantify parametric diversity.

11) Section 3.4/Figure 5 is not clear in terms of what the number of networks is and how one gets a distribution out of it?

We thank the reviewer for the opportunity to clarify our methodology for quantifying degeneracy.

We leverage the fact that all 16,038 networks are simulated using the same superset of parameter sets in 21 dimensions (corresponding to fully connected networks with 9 edges). When we simulate a network with fewer than 9 edges, the parameters corresponding to missing edges are set to zero. Conversely, when we conceptually "add" an edge to a network, the parameters for that edge come from the same 21-dimensional parameter set.

For each of the 30,000 parameter sets, we track:

1. Which network structures produce a steady state with this parameter set
2. What function does each structure exhibit

Network degeneracy is then quantified as follows: For a given parameter set and a given function, we count how many different network structures can produce that same function with that exact parameter set (where parameters for added/removed edges are the only differences).

We acknowledge that changing an edge from activation to inhibition (or vice versa) alters the structure of the ODE. In our analysis, when an edge sign changes, we maintain the corresponding K and n parameters unchanged (in the modified ODE), meaning that an activation threshold becomes an inhibition threshold with the same value. This represents a specific type of structural change where the "strength" of regulation remains the same, but its sign flips.

Figure 6 shows that the median number of structurally distinct networks that can produce the same function with the same parameter set is 60. This means that, on average, one can make substantial structural modifications (adding, removing, or changing edges) to a circuit without losing its function, provided the parameters for unchanged edges remain constant and parameters for new/removed edges follow our sampling scheme.

12) Could the core functions also be explained in terms of the effective edge from input to output, separating the case with coherent/incoherent edges as well?

We thank the reviewer for this insightful question about analysing core functions in terms of effective input-to-output paths and distinguishing coherent versus incoherent feed-forward motifs. This represents a future direction for a deeper mechanistic understanding.

Our work establishes the necessary foundation by: (1) identifying all 20 achievable functions, (2) mapping which structures produce which functions, (3) providing consensus architectures for each function (main text for top 5; Supplementary Information for all 20), and (4) developing quantitative metrics (SD, PD, FD) for systematic comparison. Future work can leverage this foundation to systematically address coherent/incoherent path analysis for functions of specific biological or synthetic interest.

Our manuscript presents an exhaustive enumeration of 16,038 structures, ~190 million circuit simulations, characterisation of 20 functions, the UNFOLD framework, and analysis of robustness, plasticity, evolvability, and canalisation. Adding a comprehensive structural decomposition of all 20 functional clusters would exceed the scope of a single publication.

Each of the 20 functions is exhibited by hundreds to thousands of distinct network structures. For example, the most common functions (FID01: exponential decay and FID02: exponential growth to saturation) are each produced by over 98% and 90% of the 16,038 networks, respectively. A thorough structural analysis would require:

4. Systematic path decomposition: Identifying all direct and indirect paths from input to output, classifying them as coherent or incoherent, and determining which path combinations are necessary for observed dynamics.
5. Parameter-dependent path dominance: The same network structure produces multiple functions depending on parameters (Section 3.3). Effective regulatory paths depend critically on kinetic parameters (thresholds K, cooperativities n, time constants τ), not topology alone. A complete analysis would require mapping how parameter variations shift dominance between competing regulatory paths.
6. Emergent network-level effects: Three-node networks exhibit complex dynamics that cannot be reduced to simple input-output paths. Our analysis revealed that adaptation can be achieved without canonical negative feedback loops or incoherent feedforward loops (Supplementary Information Section S1.2), indicating that emergent dynamics resulting from network-level interactions play a crucial role in this process.

This analysis constitutes a distinct research problem requiring:

- Motif enumeration and classification algorithms
- Dynamical systems and control theory approaches
- Comparative structural analysis frameworks to identify essential versus dispensable architectural elements

We have added a note in the Discussion identifying this as valuable future work.

## Reviewer 2

Chakraborty et al. presents a conceptual study where they 20 functions to characterize a 3 node gene circuit upon simulating over 190 million gene circuits. Their study seems to be inspired by previous studies Refs. 10,11.to study the whole design space of a 3 gene circuit and potentially finding all functional canalizations although one can argue that whether these biological functions are relevant in biology. Their framework takes the temporal information associated with a 3 node gene circuit and outputs the functional characterization clusters for the investigated circuit named "UNified FramewOrk for reguLatory Dynamics" (UNFOLD). The study is quite relevant to the challenges faced in the field however, the fact that there is no experimental data as well as the data generated is not generated by having a specific biological network in mind, I am not sure how that would perform in a real setting. I don't think this study is suitable for PLOS Computational Biology.

We thank the reviewer for their comments and welcome the opportunity to clarify the scope, methodology, and relevance of our work.

We wish to clarify a potential misunderstanding: we do not characterise a single three-node gene circuit. Rather, we systematically analyse all 16,038 possible three-node network structures, each evaluated across approximately 30,000 parameter sets, yielding over 190 million distinct genetic circuits (each representing a unique structure-parameter combination). This comprehensive coverage of the design space is central to our contribution, distinguishing our work from targeted studies of individual circuits.

UNFOLD is not a clustering tool but a unified conceptual and computational framework for analysing four fundamental biological properties—robustness, plasticity, functional canalisation, and evolvability—through three quantitative measures: structural diversity (SD), parametric diversity (PD), and functional diversity (FD). The clustering pipeline is merely one component used to systematically map the structure-parameter-function landscape.

While our simulation methodology draws inspiration from references 10 and 11 for data generation, our approach diverges fundamentally in scope and purpose. Those studies sought specific network topologies exhibiting particular functions (e.g., perfect adaptation). In contrast, we employ an unsupervised, data-driven approach to characterise the entire functional landscape without predefining or filtering for specific behaviours. Both our computational pipeline for functional classification and the UNFOLD framework itself represent novel methodological and conceptual contributions.

Regarding the question of whether our 20 functions are biologically relevant: our identified functions include well-established biological behaviours such as exponential growth to saturation, decay, oscillations, and adaptation—all documented in natural gene regulatory

systems. Notably, our finding that ~55% of circuits exhibit stabilising responses (exponential decay or growth to saturation) strongly aligns with the observed tendency of biological systems toward homeostasis. In Section 4, we explicitly discuss biological examples, including the *lac* operon, *kai* circadian genes, and the RAS gene family, demonstrating how our framework applies to characterised systems. The value lies not just in identifying these functions but in comprehensively mapping which structures and parameters can achieve them. This is essential for both understanding natural circuits and engineering synthetic ones.

We respectfully disagree that the absence of immediate experimental validation makes this work unsuitable for PLOS Computational Biology. Our work exemplifies the strength of computational biology: leveraging computational resources to establish foundational knowledge at a scale (190 million circuits) that would require prohibitively high time and resources to acquire experimentally. This bird's-eye view of the complete design space provides:

1. Fundamental limits on what three-node genetic circuits can achieve
2. A reference framework for understanding natural circuits
3. Design principles for synthetic biology applications
4. Specific, testable predictions for experimental validation (as noted in our Discussion)

Computational biology has long been recognised as encompassing both predictive modelling of specific systems and exploratory theoretical work that establishes design principles—our work firmly falls in the latter category. The comprehensive data repository we provide (publicly available on GitHub) serves as a valuable resource for the synthetic biology community, enabling rational circuit design informed by complete structure-parameter-function relationships.

We believe this systematic, large-scale computational analysis addresses fundamental questions about biological regulation that cannot be efficiently tackled through experimental approaches alone, making it well-suited for PLOS Computational Biology.

I list my major concerns below and followed by my minor concerns:
Major points:
1. If Ref. 10 is simply focusing on one function and the authors believe that their method goes far beyond what they achieved in their setting, then it would be very natural to expect that they can back out that one function cluster across the parameters space provided in Ref. 10. The same holds true for Ref. 13 in the sense of the limited ability of the tool not to properly represent certain biological setups due to restrictions on their kinetic parameters. It would be very valuable to see the failure mode of Ref. 13 is where UNFOLD shines.

We would like to clarify that we have already performed this validation analysis in Section S1.2 of the Supplementary Information. While reference 10 focused on enzyme regulatory networks

with Michaelis-Menten kinetics, our work builds on reference 11, which extended the approach to transcriptional networks using Hill kinetics—the same data generation framework we employ.

Importantly, we have already analysed adaptive networks in our dataset using the filtering criteria from references 10 and 11, as detailed in Section S1.2 of the Supplementary Information. We find that the filtering criteria used in reference 11 for detecting adaptation are not adequate because they fail to filter out responses that are oscillatory. We went one step further and applied the filtering criteria used in reference 10, finding an average of 391 networks that exhibit adaptation. We note that both references 10 and 11 had performed their analysis over 10,000 parameter sets only. However, in our case, we have 3 sets of 10,000 parameter sets (versions V0, V1, V2), which allowed us to validate the adaptive circuit count and its structural characteristics three times. We identified 97 networks that consistently demonstrated adaptation across the three versions, while the remaining structures exhibited adaptation depending on the parametric conditions. Our analysis not only identifies adaptive circuits but also challenges and extends the findings of reference 11: we discovered networks exhibiting adaptation even in topologies lacking the structural features (negative feedback loops or incoherent feedforward loops) that reference 11 claimed were necessary. This demonstrates that our framework successfully captures adaptation while revealing that the structural requirements for this function are less restrictive than previously thought. We also note that none of the structures exhibited adaptation for more than 10 parameter sets. This explains why the 20 functional clusters we found do not include adaptation, since we look for functions that are exhibited by more than 10 parameter sets (a filtering criterion that was previously used in reference 10 for enzyme regulatory networks). Thus, we have indeed backed out the adaptation function as the reviewer suggests, and our framework has provided new insights that go beyond the existing literature.

While we address the technical limitations of the Design Space Toolbox (reference 13) below, we emphasise here that UNFOLD's key advantage lies in its ability to move beyond steady-state abstractions to encompass the full dynamical landscape of gene regulatory circuits. The Design Space Toolbox (DST) is an analytical, phenotype-centric framework for rigorously exploring steady-state biochemical phenotypes, eliminating the need for prior knowledge of parameter values. DST excels in providing mechanistic, parameter-independent boundaries and symbolic understanding of system behaviour for individual systems, which is essential for targeted synthetic biology design and detailed mechanistic insight. However, UNFOLD surpasses DST by utilising an empirical, large-scale dynamical approach that systematically simulates and classifies the full temporal response behaviours across all possible three-node gene regulatory circuits and parameter sets. This enables UNFOLD to capture complex, emergent, and multifunctional dynamical properties—including degeneracy, plasticity, and stability biases—that lie beyond DST’s steady-state, analytic formalism. Thus, while DST offers unparalleled analytic precision for dissecting specific systems, UNFOLD complements and

extends beyond it by mapping the entire global structure–parameter–function space with unprecedented breadth and realism, providing a comprehensive understanding of the genetic circuit design space. We have now elaborated on the limitations of the DST for our problem in Section 1 of the revised manuscript.

2. The study seems to be quite attached to Ref. 10 and 11. Even the choice of Hill Kinetics is referenced back to Ref. 10 and 11. I wonder why Hill Kinetics is the choice where they would like to map out all biological functions.

We appreciate this question as it allows us to clarify an important distinction: our study aims to map all functions achievable by three-node *transcriptional regulatory* circuits under Hill kinetics, not all biological functions broadly. This focused scope is both intentional and appropriate for the chosen modelling framework.

Hill kinetics is the established standard for modelling transcriptional regulation because it accurately captures the cooperative binding dynamics between transcription factors and DNA that underlie gene regulation. This is not merely a methodological inheritance from references 10 and 11, but rather the biologically appropriate choice for our system. Hill kinetics is the most widely used framework in systems and synthetic biology for modelling transcriptional regulation, as evidenced by its adoption in foundational works on genetic circuits, such as references 20, 21.

Our choice is validated by extensive prior work in the field. Hill kinetics has been successfully used to model a wide range of well-characterised genetic circuits, including the lac operon, lambda phage switch, and synthetic oscillators. This choice naturally accommodates key regulatory phenomena such as cooperativity, bistability, and oscillations—all of which are experimentally observed in gene regulatory networks.

We would also note that no single modelling framework can capture 'all biological functions.' Our work provides a comprehensive map of what three-node transcriptional regulatory circuits can achieve under Hill kinetics, which represents a substantial and biologically relevant subset of regulatory behaviours. Extensions to other regulatory mechanisms (e.g., post-translational modifications, RNA-level regulation) would require different modelling frameworks although our computational pipeline can be utilised as long as the processing involves time course data. This represents important future directions, as we note in the Discussion.

3. 6. It is not clear to me what exactly fails in the parameter choices in Design Space Toolbox V3 (ref. 13) that prevents it from modeling the underlying model mechanism.

Reference 13 uses Generalised Mass Action (GMA) kinetics with power-law formulations. While mathematically flexible, GMA kinetics has two key limitations for our large-scale design space exploration:

1. Parameter interpretability: GMA parameters (kinetic orders and rate constants) are phenomenological fitting parameters that lack direct biological meaning. In contrast, Hill kinetics parameters—activation/inhibition thresholds (K), cooperativity (n), and degradation rates ($\tau$)—correspond to measurable biochemical properties. This interpretability is crucial when exploring 30,000 parameter sets across 16,038 network structures, as it allows us to understand *why* certain parameter ranges produce specific functions.
2. Numerical stability during large-scale parameter exploration: GMA's power-law formulation does not inherently enforce boundedness of gene expression levels. During extensive parameter sampling, this can lead to numerical instabilities and biologically unrealistic unbounded growth, particularly problematic when exploring diverse network topologies. Hill kinetics naturally incorporates saturation through its sigmoidal form, ensuring bounded responses that reflect biological reality—transcription factors cannot activate gene expression beyond the maximal expression level.

These are not merely theoretical concerns, but they directly impact our ability to reliably simulate and classify 190 million circuits. Hill kinetics provides the right balance of biological realism, mathematical tractability, and numerical stability for systematic design space exploration at this scale. We have better explained the limitations of the Design Space Toolbox V3 in our revised manuscript (see Section 1).

Minor points:
1. 1. Parameter sampling is a tricky word to use as there is no explicit mention of what cost function or “likelihood” they have. This is purely simulation-based study. IT is more of “parameter generation with a sampling scheme”.

We acknowledge this terminological distinction. In statistical inference contexts, 'parameter sampling' typically refers to sampling from a distribution based on observed data. Our work does not involve parameter inference or optimisation against experimental data—we systematically explore the parameter space using Latin Hypercube Sampling to characterise the full range of possible circuit behaviours.

However, 'parameter sampling' is standard terminology in computational systems biology for design space exploration studies, as evidenced by its usage in references 10, 11, 12, 13, and numerous other works in the field. We use it in this established sense—systematic generation of parameter sets to explore the design space.

2. The explanation regarding the design space being primed to produce circuits that stabilize over time is not clear to me. Especially, how they prune their networks.

We thank the reviewer for pointing out this lack of clarity. We simulate each network structure over 30,000 parameter sets. For each parameter set, we first determine the pre-stimulus steady state (with input = 0.06). We then discard parameter sets for which: (1) no steady state exists, or (2) the steady-state concentration is negligible (<0.001). This ensures that each analysed circuit is initially at a biologically relevant steady state before the input stimulus is applied. The dynamics we analyse, therefore, represent the circuit's response to perturbation, not inherent instability.

Our finding that more than 55% of circuits exhibit exponential decay (28.36%) or exponential growth to saturation (27.37%) reflects an emergent property of the design space, not an artefact of our pruning procedure. This bias toward stabilising responses appears to arise from the interplay between Hill kinetics and the parameter ranges we explored, which are based on biologically realistic values from the literature (K: 0.001-1, n: 1-4, $\tau$: 1-100). We note that this stabilising tendency aligns with biological intuition—regulatory networks in nature typically need to return to equilibrium after perturbations. In contrast, oscillatory responses (0.02% of circuits) and other complex dynamics are rare, requiring specific combinations of network structure and finely-tuned parameters. Beyond filtering parameter sets for which a given three-node network does not achieve a steady state, we have not done a ‘pruning’, which generally refers to the removal of nodes or edges of a big network.

## Reviewer 3

The paper presents a simulation framework for studying the possible functions of three-node regulatory networks. The authors simulated the dynamics of all possible circuit structures for an extensive range of parameter values, and they identified roughly 20 different circuit "functions" from unsupervised clustering of the shapes of their output dynamics.

The approach and results are very interesting for the field and seems a great fit for PLOS Computational Biology, and I personally see myself utilizing the code provided with the paper. However, the conclusions put forth in the paper, in particular that only 20 functions exist for 3 node circuits, and each can perform between 2-17 of them, are not entirely warranted by the study and should be further qualified and/or toned down for the paper to be published. I give specific comments and questions below.

The paper needs to discuss the possible sensitivity to the parameter ranges analyzed in the study as the quantitative conclusions may depend on this. In particular, the fact that the input is a step function might explain (in part) why the results are biased towards stability. Moreover, the specific parameter ranges explored, and the specific methods for comparing time-series and clustering them (and their hyperparameters), may influence the number of functions identified. While I understand that a full-fledged sensitivity analysis may be beyond the scope of the current paper due to the computational work it would involve, a partial sensitivity analysis would greatly strengthen the conclusions. This could be done at relatively little additional computational expense by rerunning the analysis on a subset of the parameter ranges to see how this influences results and by running a single additional numerical experiment with different input parameters (i.e., changing the step size and/or the parameters K_I and n_I). At the very least it should be mentioned in the abstract and conclusion that the quantitative results may depend on this and discussed how in the text.

We thank the reviewer for this important observation regarding sensitivity to our methodological choices. Our parameter ranges and the choice of Latin Hypercube Sampling are designed to provide comprehensive coverage of biologically realistic values: activation/inhibition thresholds ($K$) span three orders of magnitude (0.001-1), gene product half-lives ($\tau$) span two orders of magnitude (1-100), and Hill coefficients ($n$) range from 1-4 (integers only, to avoid numerical issues with fractional exponents in ODE solvers). These ranges align with those used in reference 11.

The reviewer raises an important point about whether our choices of input and parameter ranges may contribute to the observed stabilisation bias. We emphasise that we do not impose stability through model constraints: we use nonlinear Hill kinetics without linearisation and do not constrain parameters to ensure negative eigenvalues. However, we acknowledge that our

methodological choices—particularly the sustained step input and specific parameter ranges—may influence which dynamics are most prevalent. The >55% circuits showing stabilisation bias likely reflect a combination of (1) the intrinsic properties of Hill kinetics within biologically realistic parameter ranges, and (2) the tendency of sustained perturbations to favour circuits that return to steady state.

However, we acknowledge that our specific choices may influence the quantitative results:

1. Step input: A step function represents a sustained perturbation, which indeed may favour circuits that return to a steady state. Different input types (pulses, oscillatory inputs, ramps) might reveal additional dynamics. We have noted this as an important avenue for future work in the Discussion.
2. Parameter ranges: While our ranges are biologically motivated, they represent a particular sampling of the possible parameter space. Extending to broader ranges (e.g., $\tau$: 0.1-1000) might reveal additional rare functions.
3. Clustering methodology: We employed the DTW distance with K-means clustering and elbow-point determination to computationally determine the optimal cluster number within a range of 2 to 100. We chose this range for two reasons: (a) computational feasibility given the study's scale, and (b) coarse-graining to avoid distinguishing between functions that are only marginally different into separate clusters. We validated the robustness of our 20-function classification across three independent parameter set versions (V0, V1, V2), with 17 out of 20 functions appearing consistently in the three versions. This validation provides confidence in our main conclusions; however, we acknowledge that different distance metrics or clustering algorithms may marginally affect the function count.

A full sensitivity analysis across all parameter ranges and input types would require substantial additional computational resources (potentially multiplying our current 190 million circuit simulations several-fold). Although we agree that this would strengthen our conclusions, it is beyond the scope of the current work. We plan for future work that will extensively analyse the parameter space we have explored using both analytical and graph neural network approaches. We aim to uncover further insights into parameter space-dependent design principles for genetic circuits in our next work. In the revised manuscript, we mention that our results are valid within the scope of the parameter space and input used in both the Abstract and Discussion sections.

Similarly, the results and conclusions of the second part of the study (Section 3.5) seem highly sensitive to the particular choice of "diversity" measures defined here. These measures thus require more justification. While the definitions of SD and PD are rather straightforward (although other distance measures might be considered for PD), the definition for FD is less intuitive. In particular, why does a dissimilarity measure between a pair of circuits include a

measure of difference between all circuits with the same structure? Furthermore, why is it necessary to use a Hamming-type distance between the circuit functions, would it not be more natural to directly use DTW distances?

What is the rationale behind clustering the 20 circuit functions into the 5 (or 6) super-classes of functions and how does this influence subsequent results and conclusions? Similarly, what is the justification for the weights chosen in Supplementary Table S4, and how does their choice influence conclusions?

We thank the reviewer for this insightful question about our diversity measures. We address each component below:

UNFOLD was designed to enable the systematic analysis of biologically meaningful properties (robustness, plasticity, evolvability, canalisation) across our 190-million-circuit dataset. This required defining diversity measures that are: (1) computationally tractable for large-scale pairwise comparisons, (2) biologically interpretable, and (3) aligned with our goal of identifying discrete regions in the conceptual space corresponding to specific biological properties.

Structural Diversity (SD): Hamming distance between adjacency matrices naturally quantifies the number of regulatory edge changes (additions, removals, or sign flips), which corresponds directly to mutational events. This measure is both intuitive and standard in network comparison.

Parametric Diversity (PD): Euclidean distance in the 21-dimensional parameter space provides a straightforward measure of parametric changes. While other distance metrics (such as Manhattan and Mahalanobis) could be considered, Euclidean distance effectively captures the magnitude of parametric changes in the parameter space. We chose simplicity and interpretability over more complex metrics. Furthermore, we have checked a subset of our dataset specifically containing the parametric diversity among circuits that share the same structure, i.e., all circuit pairs that fall on the SD = 0 plane. For a given network, we considered the pairwise distance between all circuits sharing this network structure and calculated the coefficient of variation. We found that for each of the 16038 networks, the coefficient of variation is at least 0.466. This indicates that our dataset for analysing robustness (SD = 0) is not affected by the curse of dimensionality. We acknowledge that to avoid the curse of dimensionality, alternative distance metrics can be explored in future work to quantify parametric diversity.

Functional Diversity (FD): We appreciate the reviewer's note that this measure is less intuitive. FD serves a different purpose than DTW distance. While DTW measures time-series similarity (which we use in our clustering pipeline), FD quantifies the *evolutionary/mutational distance* between circuit functions in UNFOLD's framework.

FD has two components:

1. Structural component ($HD_{k\text{-hot}}$): Measures how different the functional repertoires of two network structures are. Since each network can perform multiple functions (multifunctionality), we encode this as a k-hot vector indicating which of the 20 functions it can perform. $HD_{k\text{-hot}}$ captures structural influence on multifunctionality.
2. Parametric component ($w_{ij} \times HD_{1\text{-hot}}$): Measures the categorical distance between the specific functions exhibited by two circuits. $HD_{1\text{-hot}}$ is binary (0 if same function, 1 if different), and the weight w_ij reflects the biological significance of the transition. For example, transitioning between two monophasic functions is weighted less than transitioning to oscillatory behaviour, which requires fundamentally different network properties.

The reviewer suggests using the DTW distance for FD. While DTW naturally measures time-series similarity, we deliberately chose a discrete formulation because:

Discrete FD enables clear boundaries in the conceptual space (Fig. 6) that define biological properties. For example, FD = 0 precisely demarcates the functional canalisation plane, while FD ≠ 0 defines evolvability. A continuous DTW-based measure would blur these boundaries.

Our discrete measure captures whether circuits perform the same function (FD = 0), function in the same category (FD = 0 with $w_{ij} = 0$), or belong to different functional categories (FD > 0 with varying $w_{ij}$). This categorical structure aligns with how biologists conceptualise functional similarity.

Although computing the DTW for all possible pairs of 190 million time courses is computationally expensive, it remains a tractable task. The discrete formulation, however, enables efficient querying of specific regions in the SD-PD-FD space corresponding to biological properties of interest.

We acknowledge that our specific choices of weights in Supplementary Table S4 are assigned based on the difference in phase complexity between function categories, which represents one reasonable formulation.

- $w$ = 0: Transitions within the same category (e.g., monophasic growth to monophasic decay)
- $w$ = 0.5: Transitions between categories differing by one complexity level (e.g., monophasic to biphasic)
- $w$ = 1.0: Transitions across multiple complexity levels (e.g., monophasic to oscillatory)

This weighting scheme encodes the principle that greater differences in dynamical complexity represent larger functional distances. We acknowledge that alternative schemes are possible (e.g., linearly scaling weights by complexity difference, or using uniform weights); however, qualitative patterns (e.g., which structures are most canalised, relative proportions of robust vs. plastic circuits) would remain consistent across reasonable weight variations.

In summary, our diversity measures represent a deliberate design choice optimised for UNFOLD's purpose: systematic, interpretable, and computationally tractable analysis of biological properties across the complete design space. The discretisation of FD is a feature that enables the framework. We have clarified this design rationale in the revised manuscript (Section 3.5).

Add more details on how the networks (circuit structures) are sampled. It was not clear to me from the text how this was done. In particular, whether the sampling was done with or without replacement. Related to this, the authors state that their sampling strategy allows to check for recurring patterns in the networks' temporal responses. Please explain how this is done, and why it is needed.

Why is the set of networks divided into 10 parts for the analysis? Since the results are combined afterwards and no cross-validation is performed, I do not understand why this is necessary.

We thank the reviewer for requesting clarification on our network sampling strategy. We address each question below:

We divided the complete set of 16,038 networks into ten disjoint subsets, each containing approximately 10% (~1,604 networks) of the total. Sampling was performed without replacement, ensuring that each network appears in exactly one subset and all 16,038 networks are analysed.

The partitioning procedure works as follows: We first characterised the edge-type distribution across all 16,038 networks. Specifically, for each of the nine positions in the 3×3 adjacency matrix, we calculated the frequencies of the three possible edge types: inhibition (-1), no interaction (0), and activation (+1) (Supplementary Table S2). We then divided the 16,038 networks into ten subsets using stratified random sampling without replacement, ensuring that each subset of approximately 1,604 networks maintains the same edge-type frequency distribution as the complete network collection. This stratification guarantees that each partition is structurally representative while ensuring complete coverage—every network appears in exactly one subset.

We employed this partitioning strategy rather than analysing all networks together for two reasons:

1. Computational efficiency in clustering: Our computational pipeline performs iterative time-series clustering using DTW distance calculations. Processing all ~480 million time courses (16,038 networks × ~30,000 parameter sets) simultaneously would be computationally prohibitive. Partitioning reduces the clustering problem into manageable chunks while preserving the ability to identify all function types through the subsequent meta-analysis.

2. Validation of function discovery: By analysing ten independent, structurally representative subsets, we can verify that the functions we identify are not artefacts of a particular network sample. If the same functions emerge consistently across multiple independent partitions, this provides confidence that we have captured the true functional landscape.

The reviewer inquires about how we are verifying recurring patterns and why this verification is necessary. After processing each partition through our computational pipeline, we identify 15-16 functional clusters per partition (Fig. 3). We then pool the cluster barycenters from all ten partitions and perform a clustering iteration to identify recurring function types across partitions. Functions that appear in multiple independent partitions represent robust, reproducible dynamics rather than partition-specific artefacts. This recurring pattern analysis serves as internal validation. The fact that we consistently observe the same functions across multiple structurally representative partitions (within each version) and across three independent parameter set versions (17 out of 20 functions appearing consistently in V0, V1, and V2) strengthens our confidence that we have captured the true functional landscape rather than artefacts of particular network or parameter samples.

We have clarified this sampling strategy in the revised Methods section (Section 2) to make the procedure more transparent. We have also updated the Results section to clarify the rationale behind our partitioning strategy.

The language is sometimes imprecise to the point of being incorrect. The authors for example discuss removal/addition of edges without changes in function, but this is not what is studied, only the differences in numbers and types of functions between different circuit structures. Also, at the end of the paragraph above Section 3.5.1, it is stated that the role of changes in circuit structure and/or parameters (due to mutation or epigenetic changes) can be dissected, but since the analysis is only done at the ensemble level I do not see how one can conclude on the circuits' robustness to gradual changes. At the end of Section 3.5.1 again, one cannot really say that circuits are "robust" since the influence of changes to parameters have not directly been studied. Finally, in the discussion it is suggested that "the universal multifunctionality suggests that natural circuits may have evolved to exploit this inherent versatility", but I do not see how this study tells us anything about natural evolution besides setting limits on the functions that may be achieved. Although, the limits set by this study may help understand if evolution has indeed selected preferentially for robust and/or evolvable circuits.

We appreciate the reviewer's observation that our language may have been imprecise. We clarify our network degeneracy analysis methodology here.

What we actually do: Our analysis extends beyond simply comparing the functions that different network structures can produce. We specifically track whether the same parameter set

produces the same function in different network structures. This directly addresses whether edge additions/removals can occur without functional change, as we claim.

Methodology: We leverage the fact that all 16,038 networks are simulated using the same superset of parameter sets in 21 dimensions (corresponding to fully connected networks with 9 edges). When we simulate a network with fewer than 9 edges, the parameters corresponding to missing edges are set to zero. Conversely, when we conceptually "add" an edge to a network, the parameters for that edge come from the same 21-dimensional parameter set.

For each of the 30,000 parameter sets, we track:

1. Which network structures produce a steady state with this parameter set
2. What function does each structure exhibit

Network degeneracy is then quantified as follows: For a given parameter set and a given function, we count how many different network structures can produce that same function with that exact parameter set (where parameters for added/removed edges are the only differences).

Key distinction from simple structure-function comparison: If we were only comparing "which functions different structures can produce" (as the reviewer suggests), we would compare across all parameter sets for each structure independently. Instead, we hold the parameter set constant and ask: "How many structural variants can produce the same function with this fixed parameter set?" This directly measures degeneracy—the ability to make structural changes without functional consequences when parameters remain fixed (except for those directly tied to the changed edges).

Regarding edge sign changes: We acknowledge that changing an edge from activation to inhibition (or vice versa) does alter the ODE structure. In our analysis, when an edge sign changes, we maintain the corresponding K and n parameters unchanged (in the modified ODE), meaning that an activation threshold becomes an inhibition threshold with the same value. This represents a specific type of structural change where the "strength" of regulation remains the same, but its sign flips.

Figure 6 shows that the median number of structurally distinct networks that can produce the same function with the same parameter set is 60. This means that, on average, one can make substantial structural modifications (adding, removing, or changing edges) to a circuit without losing its function, provided the parameters for unchanged edges remain constant and parameters for new/removed edges follow our sampling scheme.

We have revised Section 3.4 to make this methodology and its distinction from simple structure-function cataloguing more explicit.

The reviewer raises two related but distinct concerns about our claims. We address each precisely:

1. **Analysis is only done at the ensemble level / cannot conclude on circuits' robustness to gradual changes**

This reflects a misunderstanding of our data structure. Our analysis is fundamentally circuit-level, but it is presented with ensemble-level summaries.

What we have actually analysed is the following*:* Every data point in UNFOLD represents a specific circuit pair: ($N_i$, $P_i$, $f_i$) and ($N_j$, $P_j$, $f_j$). For Section 3.5.1 specifically, we analyse all circuit pairs where $N_i = N_j$ (same structure, $SD_{ij} = 0$) but $P_i \neq P_j$ (different parameters, $PD_{ij} \neq 0$). For each such pair, we determine whether $f_i = f_j$ (function preserved) or $f_i \neq f_j$ (function changed).

*Example of circuit-level resolution:* Consider a specific network structure $N_1$ (say, a mutual inhibition topology). In our dataset, we have ~30,000 different parameter sets producing ~30,000 circuits with this structure. We can identify, for instance, that circuit $C_1 = (N_1, P_1, f_3)$ and circuit $C_2 = (N_1, P_2, f_3)$ maintain the same function despite parametric distance $PD_{ij} = 5.2$. It is possible to further trace all circuits within any defined PD radius around $C_1$ and determine which maintain $f_3$ (constituting a "robust neighbourhood") and which switch functions (defining the boundary of robustness).

Regarding the "gradual changes" issue, the reviewer is correct that we do not model continuous trajectories. Our sampling discretises the parameter space at 30,000 points per structure. However, this discretisation is fine enough to approximate gradual changes: the median PD between parameter sets is relatively small, given our Latin Hypercube Sampling. What we measure is robustness to finite perturbations of varying magnitude (quantified by PD), not infinitesimal perturbations. This is actually more biologically relevant—mutations cause discrete jumps in parameter space, not continuous drift.

**2. Cannot really say circuits are 'robust' since the influence of parameter changes has not been directly studied**

This criticism appears to stem from terminological confusion about what we mean by "robust."

What we mean by parametric robustness: A circuit ($N_i$, $P_i$, $f_i$) is parametrically robust if there exist other parameter sets $P_j$, $P_k$, ... such that ($N_i$, $P_j$, $f_i$), ($N_i$, $P_k$, $f_i$), ... all maintain function $f_i$ despite $PD_{ij}$, $PD_{ik}$ > PD_overall_mean. This is precisely the study of "the influence of parameter changes"—we are asking whether parameter changes (of magnitude PD) preserve the function.

We do not claim to know the complete basin of attraction around each circuit in continuous parameter space. We do not claim that all intermediate points between $P_i$ and $P_j$ maintain the

function. We sample the parameter space and observe that certain (structure, parameter) combinations consistently maintain function across parametric variations.

Section 3.5.1 defines parametric robustness operationally as the "circuit pairs for which $FD_{ij} = 0$ and $PD_{ij}$ > PD_overall_mean." This is a measurable, testable definition based on our data. When we say "circuits are robust," we mean "there exist circuit pairs satisfying this criterion." This is not speculation—it's direct observation from our structure-parameter-function dataset.

The concern may be that we have not performed continuous parameter sweeps around individual circuits to map robustness boundaries. This is correct, but computationally infeasible for 190 million circuits. Our approach—dense sampling of the parameter space and pairwise comparison—provides an alternative methodology for assessing robustness that is tractable at this scale.

We make the following amendments to the revised manuscript to address the reviewer's concerns:

1. We clarify in Section 3.5 that our analysis operates at circuit-level resolution but is summarised with ensemble statistics for interpretability.
2. We revise the language to be more precise: "circuits exhibit parametric robustness" → "we identify circuit pairs demonstrating parametric robustness, defined as maintenance of function despite parametric changes exceeding the ensemble mean."

We add a methodological note acknowledging that our discrete sampling approach approximates but does not fully capture continuous parameter space structure, while arguing that this discrete approach is biologically appropriate for modelling mutational/epigenetic changes.

The reviewer is correct to challenge our speculative language. We acknowledge that our study does not provide direct evidence about evolutionary processes or selection pressures. Our phrasing conflated two distinct claims: (1) what is *possible* given physical/chemical constraints, and (2) what *actually occurred* via natural selection.

Our work establishes the *feasibility space* for three-node genetic circuits—specifically, that every network structure we examined exhibits 2-17 functions depending on parameters. This universal multifunctionality is a constraint imposed by the physics of transcriptional regulation under Hill kinetics, not an observation about evolutionary history.

We cannot conclude that evolution "exploited" this versatility without comparative genomic data showing:

1. That natural three-node circuits switch functions more frequently than would be expected by neutral drift

2. That selection acts on multifunctionality as a trait
3. That observed natural circuits occupy regions of parameter space that maximise functional plasticity

The reviewer correctly identifies that we have merely defined the boundaries of what is achievable, not what was selected.

The reviewer's own formulation is more precise: "the limits set by this study may help understand if evolution has indeed selected preferentially for robust and/or evolvable circuits." This reframing is accurate because:

1. We establish a null model wherein if we find that natural three-node circuits (e.g., lac operon, RAS pathway modules) exhibit functions that our framework predicts to be rare (e.g., oscillatory behaviour requiring specific parameter fine-tuning), this would suggest selection for those specific dynamics rather than drift within the feasible space.
2. Natural circuits could occupy any region of the SD-PD-FD space. If empirical analysis reveals they cluster in robust regions (high PD tolerance for maintaining function), this would constitute evidence for selection favouring robustness. Conversely, clustering in plastic regions would suggest selection for evolvability. Thus, our framework provides testable predictions.
3. Our finding of ~60 median degenerate structures per function means mutations causing structural changes have a high probability of being neutral (maintaining function). This creates the *possibility* for neutral evolution within functional classes, but demonstrating this requires phylogenetic analysis of actual circuits, which we have not done.

We have replaced the speculative claim with more measured language and have updated the Discussion section in the revised manuscript.

Given the plausible sensitivity of the results, the proportion of circuit pairs that show "robustness" or parameter-dependent behavior should not be reported with 4 significant digits, 2 would be more warranted.

We thank the reviewer for the suggestion. We have corrected the mentioned quantity from four significant figures to two significant figures in the revised manuscript.

How is the steady state of a circuit evaluated? Does it require only the output to be stationary or all the products (x's)?

We thank the reviewer for this important clarification question. In our analysis, a circuit is considered to have reached steady state only when all three gene products (corresponding to nodes A, B, and C) are stationary, not just the output node C.

Two complementary approaches were employed to identify steady states for each parameter set. The primary method used is 'fsolve' to numerically solve for states where all derivatives equal zero, representing true mathematical equilibria. A candidate steady state was accepted if the derivative values for all three output nodes were less than $10^{-7}$, ensuring the system would remain unchanged at that point. When 'fsolve' failed to converge to a valid steady state, either due to solver limitations or the absence of a mathematical equilibrium, a secondary empirical approach was implemented. This involved simulating the system dynamics over the specified time interval and examining the change in concentrations over the last six time points to determine if the system had reached a practical steady state. The empirical criterion required that the change in concentration for all three output nodes across these final time points be less than 5%, indicating the system had stabilised sufficiently for practical purposes. This hybrid strategy ensured robust steady state identification by combining mathematical rigour with practical simulation-based verification, particularly valuable for complex or stiff systems where numerical solvers may struggle.

This stringent criterion is biologically appropriate because gene regulatory networks operate as coupled systems—a steady state in the output alone, while internal nodes continue to change, would not represent a true equilibrium of the circuit. We have clarified this in the Methods section (Section 2) to make our steady-state determination procedure explicit.

Add a figure (e.g. to the supplementary information) showing what the "error" curve looks like for the number of clusters for choosing the number of clusters.

We appreciate the reviewer's interest in understanding our methodology for selecting cluster numbers. We would like to clarify that we did not manually inspect elbow plots to determine the optimal number of clusters. Instead, we used the kneed library (reference 16 in manuscript) to computationally and automatically detect the elbow point from the WCSS (Within-Cluster Sum of Squares) values at each clustering step.

This computational approach was necessary because:

1. Our analysis required determining the elbow point tens of thousands of times: once for each of the 16,038 networks in the first iteration of our computational pipeline, followed by clustering of the pooled barycenter datasets in subsequent iterations, repeated across three versions of parameter sets (V0, V1, V2) — totalling over 48,000 individual elbow point determinations
2. Visual inspection of tens of thousands of elbow curves would be impractical and introduce subjective variability
3. The kneed library provides a robust, reproducible, and objective mathematical method for elbow point detection that has been validated in the literature

We have added this clarification to Section 2 (Methodology).

The nondimensionalization brings down the (maximum) parameters down to 21, but it should also be mentioned how many parameters (24?) the initial formulation of the model has.

We thank the reviewer for this suggestion. We have now explicitly stated that the initial formulation of the model has 24 parameters before nondimensionalisation reduces this to a maximum of 21 parameters for fully connected three-node networks. This clarification has been added to the revised manuscript (Section 2).

Please explain the naming convention used for the different functional forms in Fig. 4 and Table 1. For example, “fast linear decay” (F08 and F10) looks slower than “linear decay” (F07) and "slow decay” (F14), "rise to peak” (F07) looks similar to “exp. growth” (F08), and “linear growth” in F03 looks more like sublinear growth to me.

We thank the reviewer for this observation. We acknowledge that the naming of the functional forms is inherently subjective and was based on visual inspection of the barycenter curves representing each cluster. We recognise that different observers may interpret these curves differently, and indeed, some of the distinctions (such as between "fast" and "slow" decay, or "linear" versus "sublinear" growth) represent qualitative judgments rather than strict mathematical classifications.

The key contribution of our work is not the specific descriptive names assigned to each function, but rather:

1. The computational identification that three-node genetic circuits can achieve exactly 20 distinct temporal response patterns (based on objective DTW-based clustering)
2. The categorisation of these 20 functions into five biologically meaningful categories (monophasic, biphasic, triphasic, oscillatory, and complex)
3. The quantitative analysis of how these functions relate to circuit structure and parameters through our UNFOLD framework

The descriptive names (e.g., "fast linear decay," "exponential growth") are provided as convenient labels to aid discussion and interpretation. We encourage readers to refer to the actual time course plots in Figure 3 and Supplementary Table S3 for their own interpretation of these dynamics. The clustering itself is based on objective DTW distances, and the biological insights regarding robustness, plasticity, evolvability, and canalisation depend on which cluster a circuit belongs to, not on the subjective naming of that cluster.

We have added a clarifying statement in the revised manuscript (Section 3.1) to note that the function names are descriptive labels based on visual inspection, and readers should refer to the plotted time courses for direct interpretation.

I suggest to add a supplementary figure showing the distribution of SD for canalized circuits to support the discussion at the end of Section 3.5

We thank the reviewer for this suggestion. We have added the plot showing the distribution of SD for canalised circuits in the supplementary information (Figure S2).

Where does the figure of 190 million circuits studied come from? Is it from the 30,000 parameter sets for the 16k networks with duplicates removed?

We thank the reviewer for seeking this clarification. The figure of ~190 million circuits comes from simulating all 16,038 network structures across 30,000 parameter sets (16,038 × 30,000 = 481,140,000 potential circuits). However, as described in our methodology, we only retained circuits for which a steady state greater than 0.001 could be determined. Parameter sets that did not meet this criterion for a given network were excluded from further analysis. After removing these unstable/non-converging cases, we obtained approximately 190 million functionally labelled circuits for our analysis.

We have clarified this in the revised manuscript (Section 2) to make the derivation of this number more transparent.

I suggest to use G_i to refer to networks (circuit structures) instead of N_i as G is standard in the literature whereas N usually refers to the number of nodes in a network.

We thank the reviewer for this helpful suggestion. We agree that using $G_i$ is more consistent with standard notation in the network literature and avoids potential confusion with the number of nodes. We have revised the notation throughout the manuscript to use $G_i$ to refer to network/circuit structures.

Calling SD, PD, and FD "diversity” measures seems like somewhat of a misnomer as they don't measure diversity but rather dissimilarity between a pair of circuits (except FD which does include a term that measures differences in the diversity of network functions, but not directly of the circuits though).

We appreciate the reviewer's observation regarding our terminology. While we agree that SD, PD, and FD are technically pairwise dissimilarity measures, we chose the term "diversity"

deliberately to reflect the biological interpretation of these quantities within our framework. In evolutionary and systems biology contexts, "diversity" commonly refers to the degree of variation—whether genetic, phenotypic, or functional—and our measures quantify exactly this variation between circuit pairs in three critical dimensions.

However, we recognise that mathematical precision is important. We have added clarifying language in Section 3.5 to explicitly state that these measures quantify pairwise dissimilarity/distance between circuits, and that we use "diversity" to emphasise their role in measuring the extent of structural, parametric, and functional variation. We believe this maintains conceptual clarity while addressing the reviewer's concern about precise mathematical nomenclature.

- P. 6, top. Refer to Eq. 1 as "Equation 1" at the start of a sentence instead of "The Eq. 1".

We thank the reviewer for this suggestion. We have made this correction in the revised manuscript.

- P. 7, how does three times 18 barycenters make 72 barycenters?

We thank the reviewer for pointing out this typographical error. We have corrected the number to 54 in the revised manuscript.

- Eq. (2) is referenced in the text before it appears. Either remove the reference or move up the equation.

We thank the reviewer for this suggestion. We have moved equation 2 next to equation 1 so that it appears before it is referenced in the revised manuscript.

- P. 10, please clarify what behavior is referred to by "... merely 0.02% of circuits exhibit this oscillatory behavior." Purely oscillatory behavior?

Yes, we thank the reviewer for this clarification request. The 0.02% refers to circuits exhibiting purely oscillatory behaviour (function FID20) in response to the step input—that is, sustained oscillations starting from an initially stable steady state. This excludes circuits with oscillatory components superimposed on other trends (classified as "complex" dynamics) or circuits showing inherent instability. We have clarified this in the revised manuscript.
