# Supplementary material for: UNified FramewOrk for reguLatory Dynamics (UNFOLD): Dissecting robustness, plasticity, evolvability and canalisation of biological function
Source: PLoS Comput Biol. 2026 May 28;22(5):e1014289. doi: 10.1371/journal.pcbi.1014289 (PMC13252850; doi:10.1371/journal.pcbi.1014289)
Supplement: Attachment — Submitted filename: Rebuttal_Revised_manuscript.pdf [file pcbi.1014289.s005.pdf]

Dear Prof. Ahuja,

We thank the reviewers for their careful reading of the manuscript and their constructive feedback. We have addressed all comments in the revised manuscript. Below, we provide point-by-point responses, with the specific changes made to the manuscript indicated in each case. Reviewer comments are reproduced in red, followed by our responses in regular text.

Reviewer 2

Chakraborty et al. provided very clarifying responses as that relates to the scope of this work such that the study presents a large-scale theoretical simulation analysis of three-node gene regulatory networks, exhaustively exploring all 16,038 network structures across (30000 parameters) extensive parameter ranges to characterize four fundamental biological properties including robustness, plasticity, functional canalization, and evolvability. I also thank the authors for clarifying how their study relates to the previously established work and pipelines as well as their choices to describe the dynamics using Hill kinetics.

After this thorough characterization of this three-node design space, I think the community appreciates if there is a description mentioned in how the practitioners would benefit from their development. They provide this exhaustive description of the three-node design space. However, practically, given the computational approach that they developed, it is not clear what they are offering to the practitioners.

We agree that the practitioner value of the framework was not stated with sufficient concreteness. In response, we have revised the Author Summary to make explicit what a practitioner receives from this work and under what conditions they can use it.

Furthermore, it is explicitly mentioned in discussion that “Our framework provides new ways to analyze such three-gene systems and identify potential therapeutic targets by understanding their regulatory dynamics within the complete functional space” however, I am still not convinced what their suggestion is at it relates to “analyze”. Most probably, they meant by “analyze” using their biological property identifying measures. However, for one to use those measures, one must know all the underlying parameters in the network at the first place. I am still of the opinion that the authors have to tone down with what they achieved at the least in abstract, summary as well as in discussion.

We have substantively revised the relevant passage in the Discussion to: (i) replace "identify potential therapeutic targets" with the more defensible formulation of "inform the prioritisation of structural or parametric perturbations as candidate therapeutic interventions," (ii) explicitly acknowledge that applying UNFOLD diversity measures to a specific biological circuit requires

knowledge of the underlying network structure and parameter values, which must be obtained from experimental data or parameter estimation, and (iii) frame any therapeutic relevance as conditional on satisfying this prerequisite.

Just as an example: the parameters take continuous values; they can be positive and take all positive real values. When they say, given the parameters they screened “all possible gene circuits” mathematically, this is in accurate.

Our analysis is based on sampling of a continuous parameter space using Latin Hypercube Sampling across 30,000 parameter sets per network structure, and claims about which structures are non-plastic or exhibit robustness are strictly valid within this sampled space rather than the full continuous space. We have now made this explicit in the Abstract.

Reviewer 3

The authors have responded in detail to our comments and amended the paper accordingly. They have in particular responded successfully to all my comments and questions about the methodology and results supporting their conclusions, and the current version of the paper provides a clear description of their methods and how their results support their conclusions.
Additionally, their inclusion and discussion of biological circuits directly demonstrates the biological relevance of the study, even if it is purely computational, and the approach and software package should be useful to study the structure-function relationship for general biological circuits beyond genetic circuits studied here. Thus, I am happy to recommend the manuscript for publication in PLOS Computation Biology.

Minor comments:

- I recommend changing “objective DTW-based clustering” to “unsupervised DTW-based clustering” in line 200 to make the statement more precise. The objectivity of k-means clustering with elbow-based model selection is contentious and not directly related to its performance.

We agree that "unsupervised" is the more technically accurate descriptor here, as it correctly characterises the nature of the clustering approach. We have made this change.

- The study of the toggle triad and its functionally homologous circuit is great. I'd suggest adding a comparison of the circuits in terms of how often each function appears in the parameter space (percentages) to give an understanding of how they may differ quantitatively.

We agree that a quantitative comparison of the two circuits enriches the functional canalisation example and gives a more complete picture of how the circuits relate not just structurally but in

terms of the distribution of behaviours across parameter space. We have added Table 2, which provides the percentage of parameter sets producing each of the 14 shared functions for both the toggle triad (8,813 valid parameter sets) and its structurally distinct equivalent (6,951 valid parameter sets). The distributions are broadly similar across most functions. For example, FID01 accounts for 36.32% and 38.79% respectively, and FID03 for 22.6% and 23.49% confirming strong functional equivalence. Quantitative differences are observed for a subset of functions, such as FID13 (1.32% vs 2.42%) and FID18 (0.92% vs 0.23%), which may reflect differences in the structural wiring rather than any deviation in the shared functional repertoire.

- Related to the discussion on lines 469-472: Finer differences (beyond shape similarity) may be relevant even for three-node circuits, e.g., if temporality is important.

We thank the reviewer for raising this important nuance. We fully agree that amplitude and temporal structure, beyond the qualitative shape captured by normalised DTW, may carry biological significance even for three-node circuits, and we have expanded the relevant discussion accordingly. The revised text explicitly acknowledges that since all time courses were normalised to [0,1] prior to clustering, circuits with identical temporal shape but differing response amplitudes receive the same functional label, and that future work could apply DTW to unnormalised trajectories or use multivariate DTW incorporating amplitude as an explicit dimension to resolve finer distinctions where the magnitude is biologically relevant. We also discuss complementary approaches including feature-augmented clustering, multi-scale DTW, and hierarchical refinement that could be applied to capture such distinctions in future work.

- Line 488: the number of admissible circuit structures is somewhat smaller than 3^{n^2} since not all matrices are connected from input to output, so I'd add “approximately".

We agree that the stated formula represents an upper bound rather than an exact count, and "approximately" is the appropriate qualifier. We have made this correction.

We believe all comments from both reviewers have now been fully addressed. We are grateful for the rigorous review process, which has strengthened the manuscript. We hope the revised version is now suitable for publication.

Yours sincerely,
Debomita Chakraborty, Raghunathan Rengaswamy, Karthik Raman
