# Supplementary material for: UNified FramewOrk for reguLatory Dynamics (UNFOLD): Dissecting robustness, plasticity, evolvability and canalisation of biological function
Source: PLoS Comput Biol. 2026 May 28;22(5):e1014289. doi: 10.1371/journal.pcbi.1014289 (PMC13252850; doi:10.1371/journal.pcbi.1014289)
Supplement: Attachment — Submitted filename: Review_PLOS.docx [file pcbi.1014289.s002.docx]

Chakraborty et al. presents a conceptual study where they 20 functions to characterize a 3 node gene circuit upon simulating over 190 million gene circuits. Their study seems to be inspired by previous studies Refs. 10,11.to study the whole design space of a 3 gene circuit and potentially finding all functional canalizations although one can argue that whether these biological functions are relevant in biology. Their framework takes the temporal information associated with a 3 node gene circuit and outputs the functional characterization clusters for the investigated circuit named “UNified FramewOrk for reguLatory Dynamics” (UNFOLD). The study is quite relevant to the challenges faced in the field however, the fact that there is no experimental data as well as the data generated is not generated by having a specific biological network in mind, I am not sure how that would perform in a real setting. I don’t think this study is suitable for PLOS Computational Biology. I list my major concerns below and followed by my minor concerns:

Major points:

1. If Ref. 10 is simply focusing on one function and the authors believe that their method goes far beyond what they achieved in their setting, then it would be very natural to expect that they can back out that one function cluster across the parameters space provided in Ref. 10.

The same holds true for Ref. 13 in the sense of the limited ability of the tool not to properly represent certain biological setups due to restrictions on their kinetic parameters. It would be very valuable to see the failure mode of Ref. 13 is where UNFOLD shines.

2. The study seems to be quite attached to Ref. 10 and 11. Even the choice of Hill Kinetics is referenced back to Ref. 10 and 11. I wonder why Hill Kinetics is the choice where they would like to map out all biological functions.
3. 6. It is not clear to me what exactly fails in the parameter choices in Design Space Toolbox V3 (ref. 13) that prevents it from modeling the underlying model mechanism.

Minor points:

1. 1. Parameter sampling is a tricky word to use as there is no explicit mention of what cost function or “likelihood” they have. This is purely simulation-based study. IT is more of “parameter generation with a sampling scheme”.
2. The explanation regarding the design space being primed to produce circuits that stabilize over time is not clear to me. Especially, how they prune their networks.
