# Supplementary material for: UNified FramewOrk for reguLatory Dynamics (UNFOLD): Dissecting robustness, plasticity, evolvability and canalisation of biological function
Source: PLoS Comput Biol. 2026 May 28;22(5):e1014289. doi: 10.1371/journal.pcbi.1014289 (PMC13252850; doi:10.1371/journal.pcbi.1014289)
Supplement: Attachment — Submitted filename: PLOS_UNFOLD_Rev2.docx [file pcbi.1014289.s004.docx]

Chakraborty et al. provided very clarifying responses as that relates to the scope of this work such that the study presents a large-scale theoretical simulation analysis of three-node gene regulatory networks, exhaustively exploring all 16,038 network structures across (30000 parameters) extensive parameter ranges to characterize four fundamental biological properties including robustness, plasticity, functional canalization, and evolvability.

I also thank the authors for clarifying how their study relates to the previously established work and pipelines as well as their choices to describe the dynamics using Hill kinetics.

After this thorough characterization of this three-node design space, I think the community appreciates if there is a description mentioned in how the practitioners would benefit from their development. They provide this exhaustive description of the three-node design space. However, practically, given the computational approach that they developed, it is not clear what they are offering to the practitioners.

Furthermore, it is explicitly mentioned in discussion that “Our framework provides new ways to analyze such three-gene systems and identify potential therapeutic targets by understanding their regulatory dynamics within the complete functional space” however, I am still not convinced what their suggestion is at it relates to “analyze”. Most probably, they meant by “analyze” using their biological property identifying measures. However, for one to use those measures, one must know all the underlying parameters in the network at the first place. I am still of the opinion that the authors have to tone down with what they achieved at the least in abstract, summary as well as in discussion. Just as an example: the parameters take continuous values; they can be positive and take all positive real values. When they say, given the parameters they screened “all possible gene circuits” mathematically, this is in accurate.
