# Supplementary material for: UNified FramewOrk for reguLatory Dynamics (UNFOLD): Dissecting robustness, plasticity, evolvability and canalisation of biological function
Source: PLoS Comput Biol. 2026 May 28;22(5):e1014289. doi: 10.1371/journal.pcbi.1014289 (PMC13252850; doi:10.1371/journal.pcbi.1014289)
Supplement: Peer review history

# Peer review: Decision Letter 0

**DOI:** 10.1371/journal.pcbi.1014289.r001

**Authors:** Stacey D. Finley, Gaurav Ahuja

29 Sep 2025

PCOMPBIOL-D-25-00986

UNified FramewOrk for reguLatory Dynamics (UNFOLD): Dissecting Robustness, Plasticity, Evolvability and Canalisation of Biological Function

PLOS Computational Biology

Dear Dr. Raman,

Thank you for submitting your manuscript to PLOS Computational Biology. After careful consideration, we feel that it has merit but does not fully meet PLOS Computational Biology's publication criteria as it currently stands. While the reviewers appreciate the novelty and extensive scope of the UNFOLD framework, they have raised significant concerns regarding the lack of biological insight and the robustness of the conclusions<source-footnote _nghost-ng-c2077508474=“” ng-version="0.0.0-PLACEHOLDER"></source-footnote><source-footnote _nghost-ng-c2077508474=“” ng-version="0.0.0-PLACEHOLDER"></source-footnote><source-footnote _nghost-ng-c2077508474=“” ng-version="0.0.0-PLACEHOLDER"></source-footnote><source-footnote _nghost-ng-c2077508474=“” ng-version="0.0.0-PLACEHOLDER"></source-footnote><source-footnote _nghost-ng-c2077508474=“” ng-version="0.0.0-PLACEHOLDER"></source-footnote><source-footnote _nghost-ng-c2077508474=“” ng-version="0.0.0-PLACEHOLDER"></source-footnote><source-footnote _nghost-ng-c2077508474=“” ng-version="0.0.0-PLACEHOLDER"></source-footnote><source-footnote _nghost-ng-c2077508474=“” ng-version="0.0.0-PLACEHOLDER"></source-footnote><source-footnote _nghost-ng-c2077508474=“” ng-version="0.0.0-PLACEHOLDER"></source-footnote>. A major revision is therefore required to strengthen the manuscript by explicitly linking network structures to the identified functions, providing stronger justification for the methodological choices, and qualifying the main claims with a discussion of their sensitivity to the parameter space and analysis methods<source-footnote _nghost-ng-c2077508474=“” ng-version="0.0.0-PLACEHOLDER"></source-footnote><source-footnote _nghost-ng-c2077508474=“” ng-version="0.0.0-PLACEHOLDER"></source-footnote><source-footnote _nghost-ng-c2077508474=“” ng-version="0.0.0-PLACEHOLDER"></source-footnote><source-footnote _nghost-ng-c2077508474=“” ng-version="0.0.0-PLACEHOLDER"></source-footnote><source-footnote _nghost-ng-c2077508474=“” ng-version="0.0.0-PLACEHOLDER"></source-footnote><source-footnote _nghost-ng-c2077508474=“” ng-version="0.0.0-PLACEHOLDER"></source-footnote><source-footnote _nghost-ng-c2077508474=“” ng-version="0.0.0-PLACEHOLDER"></source-footnote><source-footnote _nghost-ng-c2077508474=“” ng-version="0.0.0-PLACEHOLDER"></source-footnote><source-footnote _nghost-ng-c2077508474=“” ng-version="0.0.0-PLACEHOLDER"></source-footnote><source-footnote _nghost-ng-c2077508474=“” ng-version="0.0.0-PLACEHOLDER"></source-footnote><source-footnote _nghost-ng-c2077508474=“” ng-version="0.0.0-PLACEHOLDER"></source-footnote><source-footnote _nghost-ng-c2077508474=“” ng-version="0.0.0-PLACEHOLDER"></source-footnote><source-footnote _nghost-ng-c2077508474=“” ng-version="0.0.0-PLACEHOLDER"></source-footnote><source-footnote _nghost-ng-c2077508474=“” ng-version="0.0.0-PLACEHOLDER"></source-footnote><source-footnote _nghost-ng-c2077508474=“” ng-version="0.0.0-PLACEHOLDER"></source-footnote><source-footnote _nghost-ng-c2077508474=“” ng-version="0.0.0-PLACEHOLDER"></source-footnote><source-footnote _nghost-ng-c2077508474=“” ng-version="0.0.0-PLACEHOLDER"></source-footnote><source-footnote _nghost-ng-c2077508474=“” ng-version="0.0.0-PLACEHOLDER"></source-footnote><source-footnote _nghost-ng-c2077508474=“” ng-version="0.0.0-PLACEHOLDER"></source-footnote><source-footnote _nghost-ng-c2077508474=“” ng-version="0.0.0-PLACEHOLDER"></source-footnote><source-footnote _nghost-ng-c2077508474=“” ng-version="0.0.0-PLACEHOLDER"></source-footnote><source-footnote _nghost-ng-c2077508474=“” ng-version="0.0.0-PLACEHOLDER"></source-footnote><source-footnote _nghost-ng-c2077508474=“” ng-version="0.0.0-PLACEHOLDER"></source-footnote><source-footnote _nghost-ng-c2077508474=“” ng-version="0.0.0-PLACEHOLDER"></source-footnote><source-footnote _nghost-ng-c2077508474=“” ng-version="0.0.0-PLACEHOLDER"></source-footnote>.

Please submit your revised manuscript within 60 days Nov 29 2025 11:59PM. If you will need more time than this to complete your revisions, please reply to this message or contact the journal office at ploscompbiol@plos.org. When you're ready to submit your revision, log on to https://www.editorialmanager.com/pcompbiol/ and select the 'Submissions Needing Revision' folder to locate your manuscript file.

Please include the following items when submitting your revised manuscript:

* A rebuttal letter that responds to each point raised by the editor and reviewer(s). You should upload this letter as a separate file labeled 'Response to Reviewers'. This file does not need to include responses to formatting updates and technical items listed in the 'Journal Requirements' section below.

* A marked-up copy of your manuscript that highlights changes made to the original version. You should upload this as a separate file labeled 'Revised Manuscript with Track Changes'.

* An unmarked version of your revised paper without tracked changes. You should upload this as a separate file labeled 'Manuscript'.

If you would like to make changes to your financial disclosure, competing interests statement, or data availability statement, please make these updates within the submission form at the time of resubmission. Guidelines for resubmitting your figure files are available below the reviewer comments at the end of this letter

We look forward to receiving your revised manuscript.

Kind regards,

Gaurav Ahuja, PhD

Academic Editor

PLOS Computational Biology

Stacey Finley, PhD

Section Editor

PLOS Computational Biology

**Journal Requirements:**

If the reviewer comments include a recommendation to cite specific previously published works, please review and evaluate these publications to determine whether they are relevant and should be cited. There is no requirement to cite these works unless the editor has indicated otherwise.

1) Please ensure that the CRediT author contributions listed for every co-author are completed accurately and in full.

At this stage, the following Authors/Authors require contributions: Debomita Chakraborty, Raghunathan Rengaswamy, and Karthik Raman. Please ensure that the full contributions of each author are acknowledged in the "Add/Edit/Remove Authors" section of our submission form.

The list of CRediT author contributions may be found here: https://journals.plos.org/ploscompbiol/s/authorship#loc-author-contributions

2) We ask that a manuscript source file is provided at Revision. Please upload your manuscript file as a .doc, .docx, .rtf or .tex. If you are providing a .tex file, please upload it under the item type u2018LaTeX Source Fileu2019 and leave your .pdf version as the item type u2018Manuscriptu2019.

3) Please upload all main figures as separate Figure files in .tif or .eps format. For more information about how to convert and format your figure files please see our guidelines:

https://journals.plos.org/ploscompbiol/s/figures

4) We have noticed that you have uploaded Supporting Information files, but you have not included a list of legends. Please add a full list of legends for your Supporting Information files after the references list.

**Reviewers' comments:**

Reviewer's Responses to Questions

**Comments to the Authors:**

**Please note here if the review is uploaded as an attachment.**

Reviewer #1: The manuscript introduces a computational framework titled “UNified FramewOrk for reguLatory Dynamics (UNFOLD)”, which seeks to systematically classify and elucidate the dynamic behaviors of gene regulatory networks (GRNs) under diverse conditions and structural properties. This work addresses an important and timely challenge in systems biology—understanding how different network topologies and parameter configurations give rise to distinct dynamic regimes. The approach adopted by the authors is both novel and conceptually significant, as it provides a unified methodology for analyzing and categorizing GRNs based on their dynamical characteristics rather than solely on their structural features. By integrating these aspects, UNFOLD offers a valuable tool for researchers aiming to interpret the functional implications of GRN dynamics. It is commendable that the authors have done an extensive job in analysing such a large number of networks. However, the biological insights from this exercise are largely lacking, and I request the authors to address these points:

Major comments --

1) Can the authors comment on key biological insights from their analysis? Which set of networks give rise to which properties? Please present at least some examples in the high-dimensional network topology and parameter space where a particular behavior can be mapped to a set of networks, irrespective of the parameters? Similarly, it is not clear how pairwise comparisons between circuits would help to define a global conceptual space for these properties. For instance, the authors should comment on whether the properties seen by a toggle triad (Duddu et al. J R Soc Interface 2020) are seen in any other 3-node network, and if yes, how do those topologies compare to toggle triad?

2) It can get computationally intractable to extend this study to larger networks, but how would insights from this be helpful in characterizing the properties of larger networks?

3) The main text and figures do not adequately present what kind of network designs give the particular properties mentioned. The authors have mainly referred to these in supplementary figures, while the network properties are the primary focus of this study. Thus, they should consider better visualization of their key observations as main figures.

4) The authors have used LHS sampling in 21-dimensional space and reduced the dimension when not all parameters are required. However, since LHS is not independent across dimensions, can the authors justify that reducing the dimensions would maintain the necessary properties?

5) Page 8, Line 146: When it comes to 20 distinct behaviours observed in the 3-node networks, how easy or difficult is it to identify such behaviors in larger systems where the complexity of the response can increase? Is the method scalable to more complicated types of responses too?

6) Page 12, Line 224: When the authors mention structural diversity, as the gaming distance between two circuits, how would the gaming distance be calculated? Are the circuits aligned such that they give the lowest or highest hamming distance between the adjacency matrices? Would it not be better to use some kind of isomorphism measure between the circuits, as it would be more robust than just the hamming distance between two circuits?

Minor comments --

1) Is n assumed to be the same for all regulations in equation (1)?

2) While the authors have checked for connectedness from input to output, can they also check if the connectedness is satisfied between all edges to eliminate cases like A->B and A->C, where node B does not affect the output?

3) Could the core functions also be explained in terms of the effective edge from input to output, separating the case with coherent/incoherent edges as well?

4) This statement is not clear: "The percentage of circuits that exhibit the functions corresponding to each function category is much smaller than that of networks that fall in the corresponding category."

5) Page 8, Line 152: Can the parametrised Jacobian of these networks be used as a precursor for deciding which networks are stable and which would not be stable? As the time after which a network will reach the steady state is highly dependent on the parameters, having an independent method of cross-checking would make the classification more accurate. If that is something which would not be computationally scalable, can the simulations not be run for a longer time to determine if the system indeed shows oscillations?

6) Page 11, Line 205: “We define network degeneracy as a measure of structural changes (edge addition/removal/ change in the sign of an edge) that can be made, with no subsequent parameter adjustment, without causing loss of function.” Wouldn’t the removal or the change in the sign of the edge lead to a completely different ODE structure? And instead of the parameter becoming zero, the ODE itself will not have that parameter? Could the authors please clarify this section of the results a bit better?

7) Why would the clustering give some non-overlapping set of barycenters across parameter versions? This implies that the clustering is not robust across parameter replicates and opens up the possibility that there might be more than 20 circuit functions given enough sampling. Another possibility is that this could be due to the <10 time series filtering criteria.

8) It is not clear why sampling of networks is performed (although it seems more like partitioning). Is it so that the clustering algorithm can process the data in chunks?

9) Page 5, Line 64: “Of the 19683 networks possible, 3645 networks have no direct or indirect connection going from the input to the output node. Our analysis is done on the remaining 16038 networks.”

Would the analysis have been easier to do if only the non-isomorphic networks from these networks had been chosen? That would also ensure that in the meta-analysis, the reported number of behaviours would not have been biased by the number of isomorphic networks present in the networks considered.

10) Page 12, Line 226: Euclidean distance has been shown to capture very little for larger dimensions. Could the authors comment on whether this behavior is avoided in their implementation or is inconsequential for the analysis? If not, could the authors please address how the methodology can be scaled to larger networks?

11) Section 3.4/Figure 5 is not clear in terms of what the number of networks is and how one gets a distribution out of it?

12) Could the core functions also be explained in terms of the effective edge from input to output, separating the case with coherent/incoherent edges as well?

Reviewer #2: Uploaded my comments

Reviewer #3: The paper presents a simulation framework for studying the possible functions of three-node regulatory networks. The authors simulated the dynamics of all possible circuit structures for an extensive range of parameter values, and they identified roughly 20 different circuit “functions” from unsupervised clustering of the shapes of their output dynamics.

The approach and results are very interesting for the field and seems a great fit for PLOS Computational Biology, and I personally see myself utilizing the code provided with the paper. However, the conclusions put forth in the paper, in particular that only 20 functions exist for 3 node circuits, and each can perform between 2-17 of them, are not entirely warranted by the study and should be further qualified and/or toned down for the paper to be published. I give specific comments and questions below.

The paper needs to discuss the possible sensitivity to the parameter ranges analyzed in the study as the quantitative conclusions may depend on this. In particular, the fact that the input is a step function might explain (in part) why the results are biased towards stability. Moreover, the specific parameter ranges explored, and the specific methods for comparing time-series and clustering them (and their hyperparameters), may influence the number of functions identified. While I understand that a full-fledged sensitivity analysis may be beyond the scope of the current paper due to the computational work it would involve, a partial sensitivity analysis would greatly strengthen the conclusions. This could be done at relatively little additional computational expense by rerunning the analysis on a subset of the parameter ranges to see how this influences results and by running a single additional numerical experiment with different input parameters (i.e., changing the step size and/or the parameters K_I and n_I). At the very least it should be mentioned in the abstract and conclusion that the quantitative results may depend on this and discussed how in the text.

Similarly, the results and conclusions of the second part of the study (Section 3.5) seem highly sensitive to the particular choice of “diversity” measures defined here. These measures thus require more justification. While the definitions of SD and PD are rather straightforward (although other distance measures might be considered for PD), the definition for FD is less intuitive. In particular, why does a dissimilarity measure between a pair of circuits include a measure of difference between all circuits with the same structure? Furthermore, why is it necessary to use a Hamming-type distance between the circuit functions, would it not be more natural to directly use DTW distances?

What is the rationale behind clustering the 20 circuit functions into the 5 (or 6) super-classes of functions and how does this influence subsequent results and conclusions? Similarly, what is the justification for the weights chosen in Supplementary Table S4, and how does their choice influence conclusions?

Add more details on how the networks (circuit structures) are sampled. It was not clear to me from the text how this was done. In particular, whether the sampling was done with or without replacement. Related to this, the authors state that their sampling strategy allows to check for recurring patterns in the networks’ temporal responses. Please explain how this is done, and why it is needed.

Why is the set of networks divided into 10 parts for the analysis? Since the results are combined afterwards and no cross-validation is performed, I do not understand why this is necessary.

The language is sometimes imprecise to the point of being incorrect. The authors for example discuss removal/addition of edges without changes in function, but this is not what is studied, only the differences in numbers and types of functions between different circuit structures. Also, at the end of the paragraph above Section 3.5.1, it is stated that the role of changes in circuit structure and/or parameters (due to mutation or epigenetic changes) can be dissected, but since the analysis is only done at the ensemble level I do not see how one can conclude on the circuits’ robustness to gradual changes. At the end of Section 3.5.1 again, one cannot really say that circuits are "robust” since the influence of changes to parameters have not directly been studied. Finally, in the discussion it is suggested that “the universal multifunctionality suggests that natural circuits may have evolved to exploit this inherent versatility", but I do not see how this study tells us anything about natural evolution besides setting limits on the functions that may be achieved. Although, the limits set by this study may help understand if evolution has indeed selected preferentially for robust and/or evolvable circuits.

Given the plausible sensitivity of the results, the proportion of circuit pairs that show "robustness” or parameter-dependent behavior should not be reported with 4 significant digits, 2 would be more warranted.

How is the steady state of a circuit evaluated? Does it require only the output to be stationary or all the products (x's)?

Add a figure (e.g. to the supplementary information) showing what the “error” curve looks like for the number of clusters for choosing the number of clusters.

The nondimensionalization brings down the (maximum) parameters down to 21, but it should also be mentioned how many parameters (24?) the initial formulation of the model has.

Please explain the naming convention used for the different functional forms in Fig. 4 and Table 1. For example, “fast linear decay” (F08 and F10) looks slower than “linear decay” (F07) and "slow decay” (F14), "rise to peak” (F07) looks similar to “exp. growth” (F08), and “linear growth” in F03 looks more like sublinear growth to me.

I suggest to add a supplementary figure showing the distribution of SD for canalized circuits to support the discussion at the end of Section 3.5

Where does the figure of 190 million circuits studied come from? Is it from the 30,000 parameter sets for the 16k networks with duplicates removed?

I suggest to use G_i to refer to networks (circuit structures) instead of N_i as G is standard in the literature whereas N usually refers to the number of nodes in a network.

Calling SD, PD, and FD "diversity” measures seems like somewhat of a misnomer as they don't measure diversity but rather dissimilarity between a pair of circuits (except FD which does include a term that measures differences in the diversity of network functions, but not directly of the circuits though).

- P. 6, top. Refer to Eq. 1 as “Equation 1” at the start of a sentence instead of "The Eq. 1".

- P. 7, how does three times 18 barycenters make 72 barycenters?

- Eq. (2) is referenced in the text before it appears. Either remove the reference or move up the equation.

- P. 10, please clarify what behavior is referred to by “... merely 0.02% of circuits exhibit this oscillatory behavior.” Purely oscillatory behavior?

**********

**Have the authors made all data and (if applicable) computational code underlying the findings in their manuscript fully available?**

The PLOS Data policy requires authors to make all data and code underlying the findings described in their manuscript fully available without restriction, with rare exception (please refer to the Data Availability Statement in the manuscript PDF file). The data and code should be provided as part of the manuscript or its supporting information, or deposited to a public repository. For example, in addition to summary statistics, the data points behind means, medians and variance measures should be available. If there are restrictions on publicly sharing data or code —e.g. participant privacy or use of data from a third party—those must be specified.

Reviewer #1: Yes

Reviewer #2: Yes

Reviewer #3: Yes

**********

PLOS authors have the option to publish the peer review history of their article (what does this mean?). If published, this will include your full peer review and any attached files.

If you choose “no”, your identity will remain anonymous but your review may still be made public.

**Do you want your identity to be public for this peer review?** For information about this choice, including consent withdrawal, please see our Privacy Policy.

Reviewer #1: No

Reviewer #2: No

Reviewer #3: No

[NOTE: If reviewer comments were submitted as an attachment file, they will be attached to this email and accessible via the submission site. Please log into your account, locate the manuscript record, and check for the action link "View Attachments". If this link does not appear, there are no attachment files.]

**Figure resubmission:**

While revising your submission, we strongly recommend that you use PLOS’s NAAS tool (https://ngplosjournals.pagemajik.ai/artanalysis) to test your figure files. NAAS can convert your figure files to the TIFF file type and meet basic requirements (such as print size, resolution), or provide you with a report on issues that do not meet our requirements and that NAAS cannot fix.

After uploading your figures to PLOS’s NAAS tool - https://ngplosjournals.pagemajik.ai/artanalysis, NAAS will process the files provided and display the results in the "Uploaded Files" section of the page as the processing is complete. If the uploaded figures meet our requirements (or NAAS is able to fix the files to meet our requirements), the figure will be marked as "fixed" above. If NAAS is unable to fix the files, a red "failed" label will appear above. When NAAS has confirmed that the figure files meet our requirements, please download the file via the download option, and include these NAAS processed figure files when submitting your revised manuscript.

**Reproducibility:**

To enhance the reproducibility of your results, we recommend that authors of applicable studies deposit laboratory protocols in protocols.io, where a protocol can be assigned its own identifier (DOI) such that it can be cited independently in the future. Additionally, PLOS ONE offers an option to publish peer-reviewed clinical study protocols. Read more information on sharing protocols at https://plos.org/protocols?utm_medium=editorial-email&utm_source=authorletters&utm_campaign=protocols

Attachment Submitted filename: Review_PLOS.docx

# Author comment: Author response to Decision Letter 1

**DOI:** 10.1371/journal.pcbi.1014289.r002

11 Dec 2025

Attachment Submitted filename: Consolidated Rebuttal Letter.pdf

# Peer review: Decision Letter 1

**DOI:** 10.1371/journal.pcbi.1014289.r003

**Authors:** Stacey D. Finley, Gaurav Ahuja

17 Feb 2026

PCOMPBIOL-D-25-00986R1

UNified FramewOrk for reguLatory Dynamics (UNFOLD): Dissecting Robustness, Plasticity, Evolvability and Canalisation of Biological Function

PLOS Computational Biology

Dear Dr. Raman,

Thank you for submitting your manuscript to PLOS Computational Biology. After careful consideration, we feel that it has merit but does not fully meet PLOS Computational Biology's publication criteria as it currently stands. Therefore, we invite you to submit a revised version of the manuscript that addresses the points raised during the review process.

Please submit your revised manuscript by Apr 19 2026 11:59PM. If you will need more time than this to complete your revisions, please reply to this message or contact the journal office at ploscompbiol@plos.org. When you're ready to submit your revision, log on to https://www.editorialmanager.com/pcompbiol/ and select the 'Submissions Needing Revision' folder to locate your manuscript file.

Please include the following items when submitting your revised manuscript:

* A letter that responds to each point raised by the editor and reviewer(s). You should upload this letter as a separate file labeled 'Response to Reviewers'. This file does not need to include responses to formatting updates and technical items listed in the 'Journal Requirements' section below.

* A marked-up copy of your manuscript that highlights changes made to the original version. You should upload this as a separate file labeled 'Revised Manuscript with Track Changes'.

* An unmarked version of your revised paper without tracked changes. You should upload this as a separate file labeled 'Manuscript'.

If you would like to make changes to your financial disclosure, competing interests statement, or data availability statement, please make these updates within the submission form at the time of resubmission. Guidelines for resubmitting your figure files are available below the reviewer comments at the end of this letter.

We look forward to receiving your revised manuscript.

Kind regards,

Gaurav Ahuja, PhD

Academic Editor

PLOS Computational Biology

Stacey Finley, PhD

Section Editor

PLOS Computational Biology

**Additional Editor Comments:**

I have now received the reviewers’ reports on your revised manuscript, and I am pleased to inform you that all three reviewers found the revision useful and satisfactory. Reviewer #2 and #3 has requested minor revisions; I therefore ask you to incorporate these suggestions and resubmit a revised version of the manuscript.

**Reviewers' comments:**

Reviewer's Responses to Questions

**Comments to the Authors:**

**Please note here if the review is uploaded as an attachment.**

Reviewer #1: The authors have addressed my comments satisfactorily.

Reviewer #2: The review has been uploaded

Reviewer #3: The authors have responded in detail to our comments and amended the paper accordingly. They have in particular responded successfully to all my comments and questions about the methodology and results supporting their conclusions, and the current version of the paper provides a clear description of their methods and how their results support their conclusions.

Additionally, their inclusion and discussion of biological circuits directly demonstrates the biological relevance of the study, even if it is purely computational, and the approach and software package should be useful to study the structure-function relationship for general biological circuits beyond genetic circuits studied here. Thus, I am happy to recommend the manuscript for publication in PLOS Computation Biology.

Minor comments:

- I recommend changing “objective DTW-based clustering” to “unsupervised DTW-based clustering” in line 200 to make the statement more precise. The objectivity of k-means clustering with elbow-based model selection is contentious and not directly related to its performance.

- The study of the toggle triad and its functionally homologous circuit is great. I'd suggest adding a comparison of the circuits in terms of how often each function appears in the parameter space (percentages) to give an understanding of how they may differ quantitatively.

- Related to the discussion on lines 469-472: Finer differences (beyond shape similarity) may be relevant even for three-node circuits, e.g., if temporality is important.

- Line 488: the number of admissible circuit structures is somewhat smaller than 3^{n^2} since not all matrices are connected from input to output, so I'd add “approximately".

**********

**Have the authors made all data and (if applicable) computational code underlying the findings in their manuscript fully available?**

The PLOS Data policy requires authors to make all data and code underlying the findings described in their manuscript fully available without restriction, with rare exception (please refer to the Data Availability Statement in the manuscript PDF file). The data and code should be provided as part of the manuscript or its supporting information, or deposited to a public repository. For example, in addition to summary statistics, the data points behind means, medians and variance measures should be available. If there are restrictions on publicly sharing data or code —e.g. participant privacy or use of data from a third party—those must be specified.

Reviewer #1: Yes

Reviewer #2: Yes

Reviewer #3: Yes

**********

PLOS authors have the option to publish the peer review history of their article (what does this mean?). If published, this will include your full peer review and any attached files.

If you choose “no”, your identity will remain anonymous but your review may still be made public.

**Do you want your identity to be public for this peer review?** For information about this choice, including consent withdrawal, please see our Privacy Policy.

Reviewer #1: No

Reviewer #2: No

Reviewer #3: No

[NOTE: If reviewer comments were submitted as an attachment file, they will be attached to this email and accessible via the submission site. Please log into your account, locate the manuscript record, and check for the action link "View Attachments". If this link does not appear, there are no attachment files.]

**Figure resubmission:**

-->While revising your submission, we strongly recommend that you use PLOS’s NAAS tool (https://ngplosjournals.pagemajik.ai/artanalysis) to test your figure files. NAAS can convert your figure files to the TIFF file type and meet basic requirements (such as print size, resolution), or provide you with a report on issues that do not meet our requirements and that NAAS cannot fix.

After uploading your figures to PLOS’s NAAS tool - https://ngplosjournals.pagemajik.ai/artanalysis, NAAS will process the files provided and display the results in the "Uploaded Files" section of the page as the processing is complete. If the uploaded figures meet our requirements (or NAAS is able to fix the files to meet our requirements), the figure will be marked as "fixed" above. If NAAS is unable to fix the files, a red "failed" label will appear above. When NAAS has confirmed that the figure files meet our requirements, please download the file via the download option, and include these NAAS processed figure files when submitting your revised manuscript.

**Reproducibility:**

To enhance the reproducibility of your results, we recommend that authors of applicable studies deposit laboratory protocols in protocols.io, where a protocol can be assigned its own identifier (DOI) such that it can be cited independently in the future. Additionally, PLOS ONE offers an option to publish peer-reviewed clinical study protocols. Read more information on sharing protocols at https://plos.org/protocols?utm_medium=editorial-email&utm_source=authorletters&utm_campaign=protocols

Attachment Submitted filename: PLOS_UNFOLD_Rev2.docx

# Author comment: Author response to Decision Letter 2

**DOI:** 10.1371/journal.pcbi.1014289.r004

29 Mar 2026

Attachment Submitted filename: Rebuttal_Revised_manuscript.pdf

# Peer review: Decision Letter 2

**DOI:** 10.1371/journal.pcbi.1014289.r005

**Authors:** Stacey D. Finley, Gaurav Ahuja

4 May 2026

Dear Dr. Raman,

We are pleased to inform you that your manuscript 'UNified FramewOrk for reguLatory Dynamics (UNFOLD): Dissecting Robustness, Plasticity, Evolvability and Canalisation of Biological Function' has been provisionally accepted for publication in PLOS Computational Biology.

Before your manuscript can be formally accepted you will need to complete some formatting changes, which you will receive in a follow up email. A member of our team will be in touch with a set of requests.

Please note that your manuscript will not be scheduled for publication until you have made the required changes, so a swift response is appreciated.

IMPORTANT: The editorial review process is now complete. PLOS will only permit corrections to spelling, formatting or significant scientific errors from this point onwards. Requests for major changes, or any which affect the scientific understanding of your work, will cause delays to the publication date of your manuscript.

Should you, your institution's press office or the journal office choose to press release your paper, you will automatically be opted out of early publication. We ask that you notify us now if you or your institution is planning to press release the article. All press must be co-ordinated with PLOS.

Thank you again for supporting Open Access publishing; we are looking forward to publishing your work in PLOS Computational Biology.

Best regards,

Gaurav Ahuja, PhD

Academic Editor

PLOS Computational Biology

Stacey Finley, PhD

Section Editor

PLOS Computational Biology

***********************************************************

Reviewer's Responses to Questions

**Comments to the Authors:**

**Please note here if the review is uploaded as an attachment.**

Reviewer #2: I have no further comments. The authors have carefully revised the manuscript.

Reviewer #3: The revised version of the manuscript responds satisfactorily to all my comment as well as those of my co-referee, I believe, and I am happy to recommend its publication.

WRT to a possible press release, I think the subject matter and results are too technical of nature to benefit the general public.

**********

**Have the authors made all data and (if applicable) computational code underlying the findings in their manuscript fully available?**

The PLOS Data policy requires authors to make all data and code underlying the findings described in their manuscript fully available without restriction, with rare exception (please refer to the Data Availability Statement in the manuscript PDF file). The data and code should be provided as part of the manuscript or its supporting information, or deposited to a public repository. For example, in addition to summary statistics, the data points behind means, medians and variance measures should be available. If there are restrictions on publicly sharing data or code —e.g. participant privacy or use of data from a third party—those must be specified.

Reviewer #2: Yes

Reviewer #3: Yes

**********

PLOS authors have the option to publish the peer review history of their article (what does this mean?). If published, this will include your full peer review and any attached files.

If you choose “no”, your identity will remain anonymous but your review may still be made public.

**Do you want your identity to be public for this peer review?** For information about this choice, including consent withdrawal, please see our Privacy Policy.

Reviewer #2: No

Reviewer #3: No

# Editor report: Acceptance letter

**DOI:** 10.1371/journal.pcbi.1014289.r006

**Authors:** Stacey D. Finley, Gaurav Ahuja

PCOMPBIOL-D-25-00986R2

UNified FramewOrk for reguLatory Dynamics (UNFOLD): Dissecting Robustness, Plasticity, Evolvability and Canalisation of Biological Function

Dear Dr Raman,

I am pleased to inform you that your manuscript has been formally accepted for publication in PLOS Computational Biology. Your manuscript is now with our production department and you will be notified of the publication date in due course.

The corresponding author will soon be receiving a typeset proof for review, to ensure errors have not been introduced during production. Please review the PDF proof of your manuscript carefully, as this is the last chance to correct any errors. Please note that major changes, or those which affect the scientific understanding of the work, will likely cause delays to the publication date of your manuscript.

Soon after your final files are uploaded, unless you have opted out, the early version of your manuscript will be published online. The date of the early version will be your article's publication date. The final article will be published to the same URL, and all versions of the paper will be accessible to readers.

For Research, Software, and Methods articles, you will receive an invoice from PLOS for your publication fee after your manuscript has reached the completed accept phase. If you receive an email requesting payment before acceptance or for any other service, this may be a phishing scheme. Learn how to identify phishing emails and protect your accounts at https://explore.plos.org/phishing.

Thank you again for supporting PLOS Computational Biology and open-access publishing. We are looking forward to publishing your work!

With kind regards,

Anita Estes

PLOS Computational Biology | Carlyle House, Carlyle Road, Cambridge CB4 3DN | United Kingdom ploscompbiol@plos.org | Phone +44 (0) 1223-442824 | ploscompbiol.org | @PLOSCompBiol
